# Comparison of the efficacy and safety of SCD411 and reference aflibercept in patients with neovascular age-related macular degeneration

**DOI:** 10.1038/s41598-024-65815-6

**Published:** 2024-06-26

**Authors:** Se Woong Kang, Jaehwan Choi, Veeral S. Sheth, Agnieszka Nowosielska, Marta Misiuk-Hojlo, András Papp, David M. Brown, Jae-Ho Lee, Yoreh Barak

**Affiliations:** 1 Department of Ophthalmology, Samsung Medical Center, Sungkyunkwan University School of Medicine, Seoul, Korea; 2 Department of Ophthalmology, Kyung Hee University Medical Center, Kyung Hee University, Seoul, Korea; 3 Department of Clinical Research Design and Evaluation, SAIHST, Sungkyunkwan University, Seoul, Korea; 4 University Retina and Macula Associates, Lemont, IL USA; 5 Ophthalmology and Eye Surgery Clinic, Warsaw Eye Hospital, Warsaw, Poland; 6 Department and Clinic of Ophthalmology, Wroclaw Medical University, Wrocław, Poland; 7 Department of Ophthalmology, Semmelweis University, Budapest, Hungary; 8 Retina Consultants of Texas, Houston, TX USA; 9 SamChunDang Pharm. Co. Ltd., Seoul, Korea; 10 Department of Ophthalmology, Rambam Health Care Campus, Haifa, Israel

**Keywords:** Randomized controlled trials, Macular degeneration

## Abstract

To compare the efficacy and safety of the proposed aflibercept biosimilar SCD411 and reference aflibercept in patients with neovascular age-related macular degeneration, this randomized, double-masked, parallel-group, multicenter study was conducted in 14 countries from 13 August 2020 to 8 September 2022. Patients with neovascular age-related macular degeneration. With subfoveal, juxtafoveal, or extrafoveal choroidal neovascularization were aged 50 years or older. Intravitreal injection of SCD411 or aflibercept (2.0 mg) were administered every 4 weeks for the first three injections and every 8 weeks until week 48. The primary efficacy endpoint was the change in best-corrected visual acuity from baseline to week 8 with an adjusted equivalence margin of ± 3.0 letters. Patients were randomly assigned to receive either SCD411 (n = 288) or reference aflibercept (n = 288). A total of 566 participants (98.3%) completed week 8 of the study. The least-squares mean difference of change in best-corrected visual acuity from baseline to week 8 (SCD411—aflibercept) was − 0.4 letters (90% confidence interval =  − 1.6 to 0.9). The incidence of ocular (69 of 287 [24.0%] vs. 71 of 286 [24.8%]) and serious ocular (5 of 287 [1.7%] vs. 3 of 286 [1.0%]) treatment-emergent adverse effects were similar between the SCD411 and aflibercept groups. Immunogenicity analysis revealed a low incidence of neutralizing antibody formation in both groups. In conclusion, SCD411 has equivalent efficacy compared with reference aflibercept in patients with neovascular age-related macular degeneration and has a comparable safety profile. The results support the potential use of SCD411 for the treatment of neovascular age-related macular degeneration.

## Introduction

Vascular endothelial growth factor (VEGF) is the main target for treatment of neovascular age-related macular degeneration (nAMD); thus, intravitreal injection of agent inhibiting the activity of VEGF has become the standard treatment for nAMD^1–4^. Aflibercept, first introduced as “VEGF-trap”, is a soluble decoy receptor fusion protein approved by the US Food and Drug Administration in 2011^5^ and the European Medicines Agency in 2012^6^. Aflibercept has a biological advantage over ranibizumab because of its higher binding affinity to VEFG and longer duration of action^7,8^. However, the high cost of the original aflibercept is considered as a barrier for some patients and for some developing countries to access the treatment^9^. The original aflibercept comes off patent in 2023 in the USA and 2025 in Europe^10^.

Biosimilars are biological products that are highly similar to a reference product. They do not have clinically meaningful differences from the reference product in terms of safety, purity, and potency^11,12^. Biosimilars are relatively large and complex proteins that differ from generic drugs, which are small, simple, and well-defined structures that are easily characterized^11^.

SCD411 is a proposed aflibercept biosimilar that binds to VEGF-A and is produced in Chinese hamster ovary cells by using recombinant DNA technology. It has demonstrated similar structural characteristics, physicochemical properties, and biological activities similar to those of reference aflibercept in a series of in vitro and in vivo nonclinical studies. This phase 3 randomized clinical compared the efficacy, safety, pharmacokinetics, and immunogenicity of SCD411 with those of reference aflibercept.

## Methods

### Study design

This was a randomized, double-masked, parallel-group, multicenter phase 3 study conducted at 132 sites in 14 countries (e-Appendix 1 in Supplemental 1) from August 13, 2020 to September 8, 2022, and an interim analysis performed in August 2022. The study adhered to the International Council for Harmonization and Good Clinical Practice guidelines and the Declaration of Helsinki. The study protocol and protocol amendment were reviewed and approved by an independent ethics committee or institutional review board of each site (e-Appendix 2 in Supplemental 1) and registered at clinicaltrials.gov (NCT04480463, registered at 21/07/2020). A written informed consent was obtained from each participant prior to study participation. The study protocol, including treatment and statistical analysis methods and protocol amendment, is available in Supplemental 2. All imaging devices and the quality of the images obtained at each site were certified by the central reading center (Duke Reading Center, Duke University, NC, USA) prior to the initiation of the study.

### Participants

The inclusion criteria were patients aged 50 years or older and had active subfoveal, juxtafoveal, or extrafoveal choroidal neovascularization (CNV) due to AMD in the study eye. The lesion activity was confirmed using fluorescein angiography (FA). Participants with best-corrected visual acuity (BCVA) letters between 73 and 35 (Snellen equivalent to 20/200–20/32) using the original series of the Early Treatment Diabetic Retinopathy Study (ETDRS) charts or 2702 series number charts in the study eye were included. Participants were excluded if they had prior ocular, systemic, or surgical treatment for nAMD, including anti-VEGF agents; had a total lesion size > 30.5 mm^2^ including blood, scars, atrophy, fibrosis, and neovascularization in the study eye; had subretinal hemorrhage > 50% of the total lesion area or presence of subfoveal blood 1 disc area or more; had scar or fibrosis > 50% of the total lesion or involving the foveal center; had other causes of CNV in the study eye; or had signs of nAMD in the fellow eye that required any treatment. The full list of inclusion and exclusion criteria is provided in e-Appendix 3 in Supplemental 1.

### Intervention

An intravitreal injection, either 2.0 mg (0.05 mL) of SCD411 (SamChunDang Pharm. Co. Ltd., Seoul, Republic of Korea) or 2.0 mg (0.05 mL) of reference aflibercept (Eylea; Bayer HealthCare, Leverkusen, Germany), was administered every 4 weeks for the first three injections and every 8 weeks until week 48. Each study drug was provided as a single-use glass vial, containing 4 mg of study drugs in 0.1 mL solution. The other ingredients in the two study drugs were identical: polysorbate 20, sodium dihydrogen phosphate monohydrate, disodium hydrogen phosphate heptahydrate, sodium chloride, sucrose, and water for injection. The study drugs were stored in a refrigerator (2–8 ℃). The preparation and administration of the study drugs were conducted under controlled aseptic conditions. The study drugs were withdrawn into the syringe using a filter needle. Treatment of the fellow eye was allowed at any time during the study if the patient was newly diagnosed with neovascular AMD or had worsening neovascular AMD in the fellow eye. The fellow eye was treated with aflibercept according to the investigator’s discretion. Fellow eye treatment injections were not permitted on the same day as the study eye treatment.

### Randomization and masking

All participants were randomly assigned in a 1:1 ratio to receive SCD411 or Eylea intravitreal injections at baseline/randomization visit (day 1) using interactive response technology. Biostatistics was used to generate the randomization schedule using SAS software (version 9.4; SAS Institute Inc., Cary, NC, USA) for the interactive response technology, which was used to link the sequential subject randomization numbers to treatment codes. The participants, study site staff involved in subject management and study assessment, including visual acuity and image acquisition remained masked throughout the study, except for the intravitreal injection investigator and staff assigned to be unmasked for reporting of the interim analysis. The sponsor, the delegated contract research organization, and imaging teams were also masked to the study treatment.

### Outcomes

#### Efficacy endpoint

The result of VIEW^4^ (VEGF Trap-Eye: Investigation of Efficacy and Safety in Wet Age-related Macular Degeneration) studies indicated that the efficacy of treatment reaches a plateau at 12–16 weeks. Consequently, The Committee for Medicinal Products for Human Use in European Medicines Agency recommended establishing the primary efficacy endpoint before reaching this plateau. As a result, the primary endpoint was changes in BCVA from baseline to week 8, as measured using the ETDRS letter score or 2702 charts.

The secondary efficacy endpoints included changes in BCVA from baseline to week 52, changes in central retinal thickness (CRT) from baseline to weeks 8 and 52, changes in the CNV area from baseline to weeks 8 and 52, percentage of participants who gained at least 15 letters in BCVA at weeks 8 and 52. The masked readers at the Duke Reading Center measured retinal thickness, defined as the distance between internal limiting membrane and Bruch’s membrane using their in-house software (DRCVisualizer).

#### Safety

The safety endpoints for the study included the incidence of treatment-emergent adverse effects (TEAEs), vital signs, and laboratory test results. Other safety parameters included electrocardiogram results, slit-lamp examination, dilated fundoscopy, intraocular pressure, and vision checks.

No formal statistical analysis of the safety data was performed. TEAEs were defined as adverse effects (AEs) that were reported or worsened on or after the first dose date of the study drug through 28 days after the last dose date of the study drug. Adverse events were coded according to the Medical Dictionary for Regulatory Activities version 23.0 or above. Ocular TEAEs were separately summarized from non-ocular TEAEs. Ocular TEAEs were summarized for both the study and fellow eyes.

#### Immunogenicity

Immunogenicity analyses were performed at 0, 4, 8, 20, 36, and 52 weeks. Blood samples were collected prior to intravitreal injection of the study drug and FA assessment when performed on the same date. The presence of neutralizing antibodies (Nab) was tested when anti-drug antibody (ADA) results were confirmed to be positive. ADA concentration with a signal above the cutoff point of 10.0 ng/mL was defined as ADA positive.

#### Pharmacokinetics

Quantification of free and bound SCD411 and reference aflibercept was performed in patients who agreed to participate in the pharmacokinetic sub-study using blood samples before intravitreal injection and at 1, 3, 7, 14, and 28 days after the first (day 1) and third injections (week 8). Non-compartmental analysis on plasma concentration–time data were conducted using Phoenix WinNonlin® (version 8.3; Certara USA, Inc., Princeton, NJ, USA).

### Statistical analysis

#### Sample size

A sample size of 266 participants per treatment arm was selected because it provided at least 80% power and a 5% significance level. Considering an approximately 5% loss from randomization through week 8, the total sample size required was 560.

#### Analysis sets

The full analysis set (FAS) included all randomized subjects who received at least one injection of the study drug. The per-protocol set (PPS) included all participants in the FAS, excluding those with significant protocol deviations. The safety set included all patients who received at least one injection of the study drug. This was the analysis set for safety and immunogenicity analyses. The pharmacokinetic analysis set (PK set) was used to estimate the pharmacokinetic endpoints and consisted of a subset of subjects in the FAS who had sufficient evaluable blood samples. Participants were analyzed according to the treatment they received.

#### Primary and secondary efficacy analyses

The equivalence margin for primary efficacy analysis was determined using data presented by the following studies: MARINA (Minimally Classic/Occult Trial of the Anti-VEGF Antibody Ranibizumab in the Treatment of Neovascular Age-Related Macular Degeneration)^3^, ANCHOR (Anti-VEGF Antibody for the Treatment of Predominantly Classic Choroidal Neovascularization in Age-Related Macular Degeneration)^13^, and VIEW^4^ studies. For BCVA at week 8, a 90% confidence interval (CI) (equivalent to two one-sided test at an α of 0.05) with an equivalence margin of three letters was assessed. A mixed-effects model for repeated measures (MMRM) analysis was performed based on multiply imputed datasets. Rubin’s rules were used to combine the estimates from the MMRM analyses, and the estimated mean treatment differences at week 8, along with the corresponding 90% CIs, are presented. Equivalence was determined if the upper and lower CI limits for the difference between the treatments were within the equivalence margins. The analysis was based on both FAS and PPS.

The estimated mean treatment difference in the change in BCVA from baseline to week 52 and corresponding 90% CI from the same MMRM model used in the week 8 were determined. FAS and PPS were used for the analysis. The analysis of change in CRT from baseline as assessed using optical coherence tomography (OCT) and change in CNV area from baseline was based on the MMRM model, similar to that used for the analysis of change in BCVA from baseline, with the corresponding 95% CIs for the estimates of treatment differences at weeks 8 and 52. The percentage of participants who gained ≥ 15 letters in BCVA at post-baseline visits (week 8 and week 52) compared with baseline was summarized, and 95% CI for the proportions in each treatment arm and difference in proportions between treatment arms were presented. The 95% CI for the proportions in each treatment arm were calculated using the Clopper–Pearson method. The 95% CI for the difference in proportions between the treatment arms was based on the exact unconditional CI. The FAS was used for these analyses. Analyses were performed using SAS software (version 9.4).

### Ethics approval

All procedures performed in studies involving human participants were in accordance with the ethical standards of the institutional and/or national research committee and with the 1964 Helsinki Declaration and its later amendments or comparable ethical standards. The study was approved by an independent ethics committee or institutional review board of each site (e-Appendix 1 in Supplemental 1) and registered at clinicaltrials.gov (NCT04480463).

### Consent

A written informed consent was obtained from each participant prior to study participation.

## Results

### Study populations

**Figure 1:**
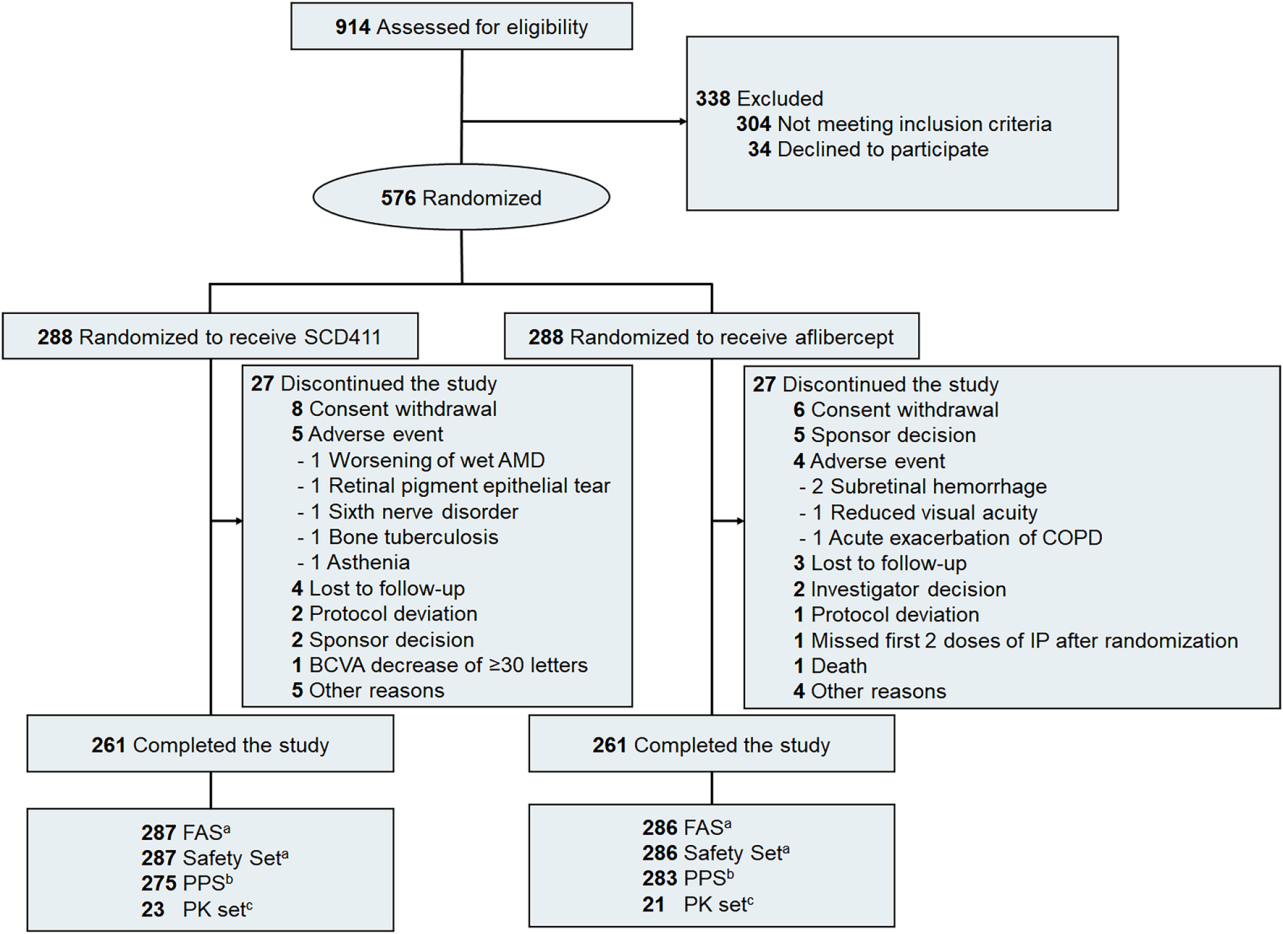
CONSORT diagram of participant disposition throughout the trial. *AMD* age-related macular degeneration, *COPD* chronic obstructive pulmonary disorder, *IP* investigational product; number, *BCVA* best-corrected visual acuity, *FAS* full analysis set, *PPS* per-protocol set, *PK* pharmacokinetics. ^a^All randomized participants who received at least 1 injection of the study drug. ^b^All participants in the FAS, excluding those with significant protocol deviations. ^C^Subset of participants in the FAS who had sufficient evaluable blood samples.

From August 13, 2020 to September 6, 2021, a total of 914 participants were screened, and 576 were randomly assigned to receive either SCD411 (n = 288) or reference aflibercept (n = 288). A total of 566 participants (98.3%) completed week 8 of the study (281 and 285 in the SCD411 and aflibercept groups, respectively). Five hundred and twenty-two participants (90.6%) completed until the end of the study (n = 261 per group). The most common reasons for discontinuing the study were withdrawal of consent from the participants (25.9%), AE (16.7%), and others (16.7%). The participants’ disposition is shown in Fig. 1.

### Demographic and ocular baseline characteristics

The baseline demographic and ocular characteristics of the participants with FAS are summarized in Table 1. Overall, the demographic and baseline characteristics were comparable between the treatment groups. The mean (SD) age of the participants was 73.5 (8.3) years. This study included 277 (48.3%) men and 296 (51.7%) women. Majority of the participants were White (382 [66.7%]) or Asian (187 [32.6%]). The mean (SD) BCVA was 59.3 (10.7) letters. The mean CRT (SD) was 490.1 (172.7) μm, and the CNV area was 4.57 (4.23) mm^2^.

**Table 1:** Baseline demographics and clinical characteristics of study participants by treatment group.

Parameters	SCD411 (N = 287)	Aflibercept (N = 286)
Age, mean (SD), y	73.5 (8.0)	73.6 (8.6)
Sex, no. (%)		
Male	138 (48.1)	139 (48.6)
Female	149 (51.9)	147 (51.4)
Race/ethnicity, no (%)		
Asian	97 (33.8)	90 (31.5)
White	188 (65.5)	194 (67.8)
Others	2 (0.6)	2 (0.6)
Weight, mean (SD), kg	72.4 (15.2)	72.3 (14.3)
Height, mean (SD), cm	164.0 (9.1)	164.6 (9.0)
BCVA, mean (SD), ETDRS letter score	58.6 (10.8)	59.9 (10.6)
CRT, mean (SD), μm	500.5 (184.0)	479.7 (160.1)
Total lesion area, mean (SD), mm^2^	5.74 (4.96)	5.28 (4.47)
CNV area, mean (SD), mm^2^	4.69 (4.29)	4.44 (4.17)
IOP, mean (SD), mmHg	15.2 (2.8)	15.3 (3.0)

*N* number, *y* year, *BCVA* best-corrected visual acuity, *ETDRS* Early Treatment Diabetic Retinopathy Study, *CRT* central retinal thickness, *CNV* choroidal neovascularization, *IOP* intraocular pressure, *SD* standard deviation.

### Efficacy endpoint results

**Figure 2:**
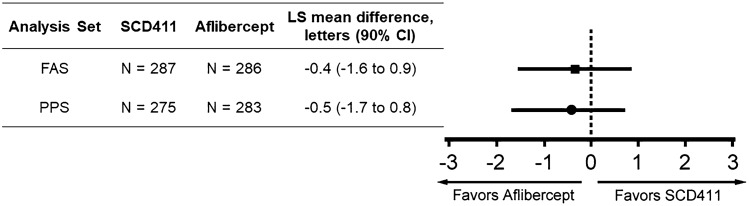
Primary efficacy results: change in best-corrected visual acuity from baseline to week 8 between the SCD411 and the aflibercept group. *LS* least squares, *CI* confidence interval, *FAS* full analysis set, *PPS* per-protocol set, *N* number.

As the primary efficacy endpoint, the least-square (LS) mean (SD) changes in BCVA score at week 8 from the baseline (FAS) were 5.5 (0.53) letters in the SCD411 group (n = 287) and 5.9 (0.52) letters in the aflibercept group (n = 286). The LS mean difference between the two groups (SCD411—aflibercept) for the estimated mean change in BCVA score from baseline was − 0.4 letters (90% CI − 1.6 to 0.9). In the PPS, a comparable result for the LS mean changes in BCVA from baseline to week 8 was also confirmed (Fig. 2).

Secondary efficacy endpoints, including BCVA score change from baseline to week 52, changes in CRT and CNV area from baseline to weeks 8 and 52, and percentage of participants who gained at least 15 letters in BCVA at weeks 8 and 52, showed similar efficacy between the treatment groups (Tables 2, 3). In addition, the changes in the BCVA score and CRT from baseline at all visits to week 52 were comparable between the treatment groups (eFigs. 1, 2 in Supplemental 1).

**Table 2:** Secondary efficacy results at weeks 8 and 52.

Parameters—analysis set	Change from baseline, LS mean (SE)		Difference (SCD411—Aflibercept)	
	SCD411	Aflibercept	Mean (SE)	95% CI (90% CI for BCVA)
BCVA at week 52 (letters)				
FAS	9.0 (0.71)	7.7 (0.71)	1.3 (1.01)	− 0.4 to 2.9
PPS	8.9 (0.72)	7.7 (0.72)	1.2 (1.02)	− 0.5 to 2.9
CRT at week 8 (μm)				
FAS	− 176.7 (5.3)	− 177.2 (5.3)	0.5 (7.48)	− 14.2 to 15.2
CRT at week 52 (μm)				
FAS	− 200.6 (5.2)	− 189.9 (5.2)	− 10.7 (7.36)	− 25.1 to 3.8
CNV area at week 8 (mm^2^)				
FAS	− 1.16 (0.14)	− 1.38 (0.14)	0.21 (0.20)	− 0.18 to 0.61
CNV area at week 52 (mm^2^)				
FAS	− 2.38 (0.16)	− 2.09 (0.16)	− 0.28 (0.23)	− 0.73 to 0.16

*LS* least squares, *SE* standard error, *BCVA* best-corrected visual acuity, *CI* confidence interval, *CRT* central retinal thickness, *CNV* choroidal neovascularization, *FAS* full analysis set, *PPS* per-protocol set.

**Table 3:** Dichotomous secondary efficacy results: proportion of participants who gained ≥ 15 letters in BCVA score from baseline to week 8 and 52.

Subjects who gained at least 15 letters in BCVA–FAS	SCD411, N (%)	Aflibercept, N (%)	Difference (SCD411—Aflibercept) (%) (95% CI)
Week 8	34 (11.8)	35 (12.2)	− 0.4 (− 5.8 to 5.0)
Week 52	86 (30.0)	66 (23.1)	6.9 (− 0.4 to 14.1)

*BCVA* best-corrected visual acuity, *N* number, *CI* confidence interval.

### Safety

Exposure was similar between the SCD411 (n = 287) and aflibercept (n = 286) groups, including the mean (SD) number of injections (7.6 [1.32] vs. 7.6 [1.14]) and mean (SD) duration of drug exposure (317.1 [68.74] vs. 318.2 [61.36]). The number of participants treated with anti-VEGF in the fellow eye was not significantly different between the SCD411 (26 [9.1%]) and aflibercept (18 [6.3%]) groups. The incidence of ocular and non-ocular TEAEs was similar between the SCD411 and aflibercept groups (ocular TEAEs, 69 [24.0%] vs. 71 [24.8%]; serious ocular TEAEs, 5 [1.7%] vs. 3 [1.0%]; and non-ocular TEAEs, 128 [44.6%] vs. 130 [45.5%]). The number of TEAEs leading to study drug discontinuation was also similar between the SCD411 and aflibercept groups (ocular TEAEs, 2 [0.7%] in both groups; non-ocular TEAEs, 2 [0.7%] vs. 1 [0.3%]). No ocular or non-ocular TEAE that led to death were observed in either group. Ocular TEAE was not noted in the study eye in ≥ 5% of the patients, and the most common ocular TEAE was reduced visual acuity (13 [4.5%] in two groups), followed by conjunctival hemorrhage (SCD411, 8 [2.8%] vs. aflibercept, 6 [2.1%]). A retinal pigment epithelial tear was reported in three (1.0%) in the SCD 411 group and two (0.7%) in the aflibercept group (Table 4).

**Table 4:** Summary of adverse events in the safety set.

Adverse event	Subjects, N (%)		
	SCD411 N = 287	Aflibercept N = 286	Total N = 573
Ocular TEAE			
Study eye			
Any TEAE	69 (24.0)	71 (24.8)	140 (24.4)
Serious TEAEs	5 (1.7)	3 (1.0)	8 (1.4)
TEAEs leading to treatment discontinuation	4 (1.4)	5 (1.7)	9 (1.6)
AESI	17 (5.9)	15 (5.2)	32 (5.6)
Fellow eye			
Any TEAE	54 (18.8)	48 (16.8)	102 (17.8)
Serious TEAEs	0 (0.0)	1 (0.3)	1 (0.2)
TEAEs leading to treatment discontinuation	0 (0.0)	0 (0.0)	0 (0.0)
AESI	9 (3.1)	9 (3.1)	18 (3.1)
Non-ocular TEAE			
Any TEAE	128 (44.6%)	130 (45.5%)	258 (45.0)
Serious TEAEs	27 (9.4)	27 (9.4)	54 (9.4)
TEAEs leading to treatment discontinuation	3 (1.0)	4 (1.4)	7 (1.2)
AESI	5 (1.7)	10 (3.5)	15 (2.6)
Any TEAE leading to death	0 (0.0)	0 (0.0)	0 (0.0)
Ocular TEAEs in the study eye			
Reduced visual acuity	13 (4.5)	13 (4.5)	26 (4.5)
Conjunctival hemorrhage	8 (2.8)	6 (2.1)	14 (2.4)
Retinal hemorrhage	1 (0.3)	5 (1.7)	6 (1.0)
Cataract	5 (1.7)	4 (1.4)	9 (11.6)
Posterior capsule opacification	4 (1.4)	1 (0.3)	5 (0.9)
Retinal pigment epithelial tear	3 (1.0)	2 (0.7)	5 (0.9)
Endophthalmitis	1 (0.3)	0 (0.0)	1 (0.2)
Ocular TEAEs in the fellow eye			
Neovascular age-related macular degeneration	20 (7.0)	13 (4.5)	33 (5.8)
Reduced visual acuity	2 (0.7)	3 (1.0)	5 (0.9)
Non-ocular TEAEs			
Hypertension	12 (4.2)	4 (1.4)	16 (2.8)
Cardiac failure	1 (0.3)	0 (0.0)	1 (0.2)
Myocadiac ischemia	0 (0.0)	1 (0.3)	1 (0.2)
Neoplasms	3 (1.0)	3 (1.0)	6 (1.0)

*N* number, *TEAE* treatment-emergent adverse event, *AESI* adverse event of special interest.

### Pharmcokinetic results

PK analysis was performed for 23 participants in the SCD411 group and 21 participants in the aflibercept group, and the results are summarized in eFig. 3 in Supplemental 1. Following a single 2-mg intravitreal injection of SCD411 or Eylea on day 1 and at week 8, the mean free SCD411 and Eylea concentration versus time profiles showed similar PK properties.

### Immunogenicity results

The cumulative incidence of ADA was similar between the two groups. ADA positivity was 7.1% in the SCD411 group and 6.9% in the aflibercept group at baseline and 20.2–40.0% in the SCD411 group and 18.8–52.3% in the aflibercept group during the study period. The incidence of Nab was low and similar between the two groups. Of the participants who provided blood samples for immunogenicity assessment at baseline, 1.1% of the participants in the SCD411 group and 2.2% of the participants in the aflibercept group were NAb-positive at baseline. The incidence of Nab-positive subjects remained < 3% in the SCD411 group and mostly remained < 4% in the aflibercept group during the study period. (eTable 1 in Supplemental 1).

## Discussion

This phase 3, randomized, double-masked, parallel-group, multicenter study demonstrated that the proposed biosimilar SCD411 had equivalent efficacy, safety, immunogenicity, and pharmacokinetic profile for the treatment of nAMD to the reference aflibercept. The primary end-point of this study was achieved, because the difference in the change in BCVA from baseline to week 8 between the SCD411 and aflibercept groups was within a predefined equivalence margin. In addition, the results of the secondary end points supported the equivalent efficacy of SCD411 and reference aflibercept. Sensitivity analyses for the primary end point also showed robust equivalence of efficacy between the two groups.

Based on the safety data of the total population, SCD411 was well tolerated, with a favorable safety profile comparable with that of reference aflibercept. The incidences of ocular and non-ocular TEAEs were similar between the two treatment groups. The mean peak plasma concentrations of free SCD411 and reference aflibercept after week 8 were 50.7 and 43.0 ng/mL, respectively and were more than 50-fold lower than the concentration of aflibercept required to inhibit the half of free VEGF activity^14^. This result was similar with those obtained by previous studies on nAMD, which ranged from 20.0 to 66.7 ng/mL in patients with nAMD^4,15^. Considering the low systemic exposure of SCD411 along with reference aflibercept, the risk of systemic adverse effect would be low. Immunogenicity assessments showed that the observed immunogenicity leves were similar between the SCD411 and aflibercept groups with a low incidence of NAb positivity.

Previous clinical trials on intravitreal anti-VEGF injections for nAMD showed that changes in BCVA and CRT rapidly coccurred within the initial 8–12 weeks of treatment and then stabilized thereafter^3,4,13,16^. In addition, the primary outcome of the VIEW studies was BCVA at week 52^4^. Therefore, assessing the clinical outcomes at weeks 8 and 52 is a reasonable approach to evaluate the efficacy of SCD411 for the treatment of nAMD.

The results of this study are consistent with those of previous aflibercept clinical trials in terms of external validity. The mean BCVA change at week 8 was 5.9 letters, and it was similar to the BCVA change in the VIEW studies, which was approximately 6 letters. At week 52, the mean change of BCVA was 7.7 letters in the aflibercept group, compared with 7.9 letters in VIEW 1, 8.9 letters in VIEW 2, and 10.2 letters in ARIES (Age-related Macular Degeneration Requiring Intensive Intravitreal Aflibercept Treatment) studies of same treatment protocol^4,17^. The mean CRT change from baseline to week 52 was − 189.9 μm, compared with − 128.5 μm in VIEW 1 and − 149.2 μm in VIEW 2 studies of same treatment protocol^4^.

Several studies have demonstrated a discrepancy between the outcomes of clinical trials of anti-VEGF agents and real-world data^18,19^. Longer-acting agents and lower treatment costs are required to overcome these compliance issues. Aflibercept has been known to exert a relatively longer duration of action; therefore, fewer clinic visits are possible^4,7,20^. A biosimilar product of aflibercept can provide additional treatment options for patients with nAMD whose condition is suitable for aflibercept and is expected to alleviate financial burdens on patients in some countries.

This study had several limitations. The number of participants in this study was sufficient to compare the equivalence in efficacy between SCD411 and the reference aflibercept, but it may not have been sufficient to observe very rare complications associated with the biosimilar product SCD411. Therefore, further large-scale studies are warranted. This study followed the participants for 52 weeks. Although there was no significant difference in the changes in the BCVA and CRT from the baseline at all study visits, the SCD411 group showed a trend towards better BCVA and CRT changes at the end of the follow-up. In this aspect, additional studies assessing long-term clinical outcomes are required.

In conclusion, the proposed aflibercept biosimilar product, SCD411, had efficacy, safety, and immunogenicity equivalent to the reference aflibercept in patients with nAMD. These results support the use of SCD411 as a promising aflibercept biosimilar.

### Supplementary Information

Supplementary Information 1.

Supplementary Information 2.

Supplementary Information 3.

## Data Availability

Subject to specific criteria, conditions, and exceptions, SamChunDang Pharm. Co. Ltd. is committed to granting researchers access to individual deidentified participant data, provided their proposals satisfy the research criteria and meet all other applicable conditions. Interested researchers are kindly requested to submit their proposals directly to the corresponding author. In order to obtain access, data requestors are required to enter into a data access agreement with SamChunDang Pharm. Co. Ltd.
