# Supplementary material for: Comparison of the efficacy and safety of SCD411 and reference aflibercept in patients with neovascular age-related macular degeneration
Source: Sci Rep. 2024 Jun 26;14:14752. doi: 10.1038/s41598-024-65815-6 (PMC11208512; doi:10.1038/s41598-024-65815-6)

**e-Appendix 1.** List of investigators and study centers

| **Investigator** | **Site number** | **Address of the study center** |
| --- | --- | --- |
| **Australia** |  |  |
| Andrew Chang | 1002 | Sydney Retina Clinic and Day Surgery,  187 Macquarie Street,  Sydney  New South Wales, 2000  Australia |
| Fred Chen | 1003 | Lions Eye Institute  2 Verdun Street  Nedlands  Western Australia, 6009  Australia |
| Thanh Nguyen | 1004 | Centre For Eye Research Australia Ltd, 32 Gisborne Street, East Melbourne, Victoria, 3002 Australia |
| Gerald Liew | 1006 | South West Retina 57-59 Memorial Avenue Liverpool New South Wales, 2170 Australia |
| **Poland** |  |  |
| Jerzy Mackiewicz | 1201 | Samodzielny Publiczny Szpital Kliniczny Nr 1 w Lublinie Klinika Chirurgii Siatkówki i Ciala Szklistego ul. Chmielna 1 Lublin, 20-079 Poland |
| Krystyna Raczynska | 1202 | Optimum Profesorskie Centrum Okulistyki ul. Cienista 30 Gdańsk, 80-809 Poland |
| Piotr Fryczkowski | 1203 | Retina Okulistyka Sp. z o.o. Sp.k. ul. Gimnazjalna 1 Warszawa, 01-364 Poland |
| Bartlomiej Katuzny | 1204 | Oftalmika Sp. z o. o. Modrzewiowa 15 Bydgoszcz, 85-631 Poland |
| Robert Leszek | 1205 | 4 Wojskowy Szpital Kliniczny z Poliklinika SP ZOZ we Wroclawiu Kliniczny Oddział Okulistyczny ul. Rudolfa Weigla 5 Wroclaw, 50-981 Poland |
| Marta Misiuk-Hojlo | 1206 | Uniwersytecki Szpital Kliniczny im. Jana Mikulicza-Radeckiego we Wrocławiu Klinika Okulistyki ul. Borowska 213 Wroctaw, 50-556 Poland |
| Ewa Mrukwa-Kiminek | 1207 | Uniwersyteckie Centrum Kliniczne im. prof. K. Gibińskiego Śląskiego Uniwersytetu Medycznego w Katowicach Oddział Okulistyki Dorostych ul. Ceglana 35 Katowice, 40-514 Poland |
| Agnieszka Nowosielska | 1208 | Warszawski Szpital Okulistyczny ul. Wolska 165 lokal U7 Warszawa, 01-258 Poland |
| Bartosz Sikorski | 1209 | Specjalistyczny Ośrodek Okulistyczny Oculomedica ul. Ogrady 14 Bydgoszcz, 85-870 Poland |
| Slawomir Teper | 1210 | Gabi net Okulistyczny Prof. Edward Wylegała ul. Gallusa 4 Katowice, 40-594 Poland |
| Dominik Zalewski | 1211 | Centrum Diagnostyki i Mikrochirurgii Oka - Lens dr n. med. Sławomir Zalewski ul. Budowlana 3A Olsztyn, 10-424 Poland |
| Joanna Gołebiewska | 1212 | Centrum Mikrochirurgii Oka Laser Klinika Profesora Jerzego Szaflika ul. Dolańskiego 2 Warszawa, 00-215 Poland |
| Justyna Mazur-Samela | 1214 | Osrodek Chirurgii Oka Prof. Zagórskiego Sp. z o. o. ul. S. Moniuszki 8 Rzeszów, 35-017 Poland |
| Tomasz Zarnowski | 1215 | Samodzielny Publiczny Szpital Kliniczny nr 1 w Lubl inie Klinika Diagnostyki i Mikrochirurgii Jaskry ul. Chmielna 1 Lublin, 20-079 Poland |
| Ewa Fluder | 1217 | Specjalistyczny Szpital im. dra Alfreda Sokołowskiego Oddział Okulistyczny ul. Sokotowskiego 4 Walbrzych, 58-309 Poland |
| **Russia** |  |  |
| Alexander Doga | 1301 | Federal State Autonomous Institution “National Medical Research Centre” “Interdisciplinary Scientific and Technical Complex” “Microsurgery of the Eye” n. a. “S.N. Fedorov" of the Ministry of Health of the Russian Federation Beskudnikovsky bulvar, 59a Moscow Moskovskaya oblast, 127486 Russia |
| Natalia Gavrilova | 1302 | State Budgetary Educational Institution of Higher Professional Education “Moscow State Medical Stomatology University n. a. A.I. Evdokimov” of the Ministry of Health of the Russian Federation, Department of Ophthalmology Clinical Medical Centre Legal address: ul. Delegatskava, 20, Street. 1 Actual address: ul.Kuskovskaya, vladeniye 1A, Street 4 Moscow Moskovskaya oblast, 127473/111398 Russia |
| Tatyana Okhotsymskaya | 1304 | Federal State Budgetary Institution "Helmholtz National Medical Research Centre of Eye Diseases" of the Ministry of Health of the Russian Federation ul. Sadovaya-Chernogryazskaya, 14/19 Moscow Moskovskaya oblast, 105062 Russia |
| Anzhella Fursova | 1305 | State Budgetary Healthcare Institution of Novosibirsk Region “State Novosibirsk Regional Clinical Hospital” Ophthalmology Department ul. Nemirovicha-Danchenko, 130 Novosibirsk Novosibirsk region, 630087 Russia |
| **Slovakia** |  |  |
| Mikuláš Alexik | 1401 | Oftalmologické oddelenie Fakultná nemocnica s poliklinikou Žilina Vojtecha Spanyola 43 Žilina, 01207 Slovakia |
| Mária Hurčíková | 1402 | Očné oddelenie - jednodňová zdravotná starostlivost Nemocnica spoliklinikou Trebiŝov, a.s., Ulica SNP 1079/76 Trebisov, 075 01 Slovakia |
| Livia Javorska | 1403 | Oftalmologické oddelenie jednodnová zdravotna starostlivost, Nemocnica Poprad, a.s. Bánicka 803/28 Poprad, 058 45 Slovakia |
| Jana Štefaničkova | 1404 | Klinika oftalmologie LFUK a UNB Univerzitná nemocnica Bratislava Nemocnica Ružinov Ružinovska 6 Bratislava, 826 06 Slovakia |
| **Spain** |  |  |
| Jose Javier Araiz Iribarren | 1501 | Instituto Clínico Quirurgico de Oftalmologia Alameda de Recalde 49-51 Bilbao Vizcaya, 48010 Spain |
| Jose Juan Escobar Barranco | 1502 | Hospital Dos de Maig Carrer del Dos de Maig, 301 Barcelona, 08025 Spain |
| Ignasi Jürgens Mestre | 1503 | Institut Catala de Retina (ICR) Calle Ganduxer, 117 Barcelona, 08022 Spain |
| Javier Montero Moreno | 1504 | Hospital Universitario Rio Hortega Calle Dulzaina, 2 Valladolid, 47012 Spain |
| Luis Pablo Julvez | 1505 | Hospital Universitario Miguel Servet Paseo Isabel La Cató1ica 1-3 Zaragoza, 50009 Spain |
| Jose Maria Ruiz Moreno | 1506 | Hospital Universitario Puerta de Hierro - Majadahonda Calle Manuel de Falla, 1 Madrid, 28222 Spain |
| Laura Sararols Ramsay | 1507 | Hospital Universitari General de Catalunya - Grupo Quironsalud Calle Pedro i Pons,1 Valles Oftalmologfa Recerca Planta Baja San Cugat del Valles Barcelona, 08195 Spain |
| Enrique Cervera Taulet | 1509 | Consorcio Hospital General Universitario de Valencia Avenida De les Tres Cruces, 2 Valencia, 46014 Spain |
| Francisco Javier Ascaso Puyuelo | 1511 | Hospital Clínico Universitario Lozano Blesa Avenida San Juan Bosco, 15 Zaragoza, 50009 Spain |
| **USA** |  |  |
| Ross Lynds  Courtney Crawford | 1601 | Strategic Clinical Research Group 101 Chuckwagon Trail Willow Park, TX 76087 USA |
| Dana M Deupree | 1603 | Blue Ocean Clinical Research 3280 N McMullen Booth Clearwater, FL 33761 USA |
| Shailesh K. Gupta | 1606 | Advanced Research LLC. 6280 W. Sample Road Suite 202 Coral Springs, FL 33067 USA |
| Sunil S. Patel | 1610 | Retina Research Institute of Texas 5441 Health Center Drive Abilene, TX 79606 USA |
| Ashish Sharma  Joseph P. Walker | 1611 | National Ophthalmic Research Institute 6901 International Center Boulevard Fort Myers, FL 33912 USA |
| Prema Abraham | 1612 | Black Hills Regional Eye Institute 2800 Third Street Rapid City, SD 57701 USA |
| Jamin Brown | 1614 | Retina Vitreous Surgeons of Central New York, PC 200 Greenfield Parkway Liverpool, NY 13088 USA |
| Amr Dessouki | 1615 | Retinal Diagnostic Center 3395 S. Bascom Avenue Suite 140 Campbell, CA 95008 USA |
| Allan A. Hunter III | 1618 | Cascade Medical Research Institute, LLC 3783 International Court Suite 290 Springfield, OR 97477 USA |
| Veeral Sheth | 1621 | University Retina and Macula Associates, PC 15947 W. 127th Street Suite E Lemont, IL 60439 USA |
| Robert A Stoltz | 1622 | Georgia Retina, P.C. 833 Campbell Hill Street Suite 300 Marietta, GA 30060 USA |
| John Randolph | 1626 | Center for Retina and Macular Disease 250 Avenue. K, S.W. Suite 200 Winter Haven, FL 33880 USA |
| **Korea** |  |  |
| Joo Yong Lee | 1701 | Asan Medical Center 88, Olympic-ro 43-gil Songpa-gu Seoul, 05505 Korea |
| Kihwang Lee | 1702 | Ajou University Hospital 164, Worldcup-ro Yeongtong-gu, Suwon-si Gyeonggi-do, 16499 Republic of Korea |
| Iksoo Byon | 1703 | Pusan National University Hospital 179, Gudeok-ro Seo-gu Busan, 49241 Republic of Korea |
| Joo Young Shin | 1704 | SMG – SNU Boramae Medical Center 20, Boramae-ro 5-gil Dongjak-gu Seoul, 07061 Republic of Korea |
| Yu Cheol Kim | 1705 | Keimyung University Dongsan Hospital 1035, Dalbugeol-daero Dalseo-gu Daegu, 42601 Republic of Korea |
| Min Sagong | 1706 | Yeungnam University Hospital 170 Hyeonchung-ro Nam-gu Daegu, 42415 Republic of Korea |
| Dong Ho Park | 1707 | Kyungpook National University Hospital 130 Dongdeok-ro Jung-gu Daegu, 41944 Republic of Korea |
| Hee Seung Chin | 1708 | Inha University Hospital 27 lnhang-ro Jung-gu Incheon, 22332 Republic of Korea |
| Se Woong Kang | 1710 | Samsung Medical Center 81, Irwon-ro Gangnam-gu Seoul, 06351 Republic of Korea |
| Seung-Young Yu | 1712 | Kyung Hee University Hospital 23, Kyungheedae-ro Dongdaemun- gu Seoul, 02447 Republic of Korea |
| Yong Seop Han | 1713 | Gyeongsang National University Changwon Hospital 11 Samjeongja-ro, Seongsan-gu Changwon-si Gyeongsangnam-do, 51472 Republic of Korea |
| Yong-Un Shin | 1714 | Hanyang University Guri Hospital 153 Gyeongchun-ro Guri-si Gyeonggi-do, 11923 Republic of Korea |
| Eun Kyoung Lee  Un Chul Park | 1715 | Seoul National University Hospital 101 Daehak-ro Jongno-gu Seoul, 03080 Republic of Korea |
| Yong Sung You | 1716 | 14F, Nune Eye Hospital 408, Teheran-ro Gangnam-Gu Seoul, 06192 Republic of Korea |
| Sung Pyo Park | 1717 | Hallym University Kangdong Sacred Heart Hospital 150, Seongan-ro Gangdong-gu Seoul, 05355 Republic of Korea |
| Yoon-Hyung Kwon | 1718 | Dong-A University Hospital 26 Daesingongwon-Ro Seo-Gu Busan, 602-715 Republic of Korea |
| Sang-Joon Lee | 1719 | Kosin University Gospel Hospital 262, Gamcheon-ro Busan, 49267 Republic of Korea |
| Young Hoon Hwang  Jung Yeul Kim | 1720 | Chungnam National University Hospital 282 Munhwa-ro Jung-Gu Daejeon, 35015 Republic of Korea |
| JaeRyung Oh | 1721 | Korea University Anam Hospital 73, Inchon-ro Seongbuk-gu Seoul, 02841 Republic of Korea |
| KyungTae Kim | 1722 | Chungbuk National University Hospital 776, Isunhwan-ro Seowon-gu, Cheongju-si Chungcheongbuk-do, 28644 Republic of Korea |
| Dae Yeong Lee | 1723 | Gachon University Gil Medical Center 21, Namdong-daero 774 beon-gil Namdong-gu Incheon, 21565 Republic of Korea |
| In Young Chung | 1724 | Gyeongsang National University Hospital 79, Gangnam-ro, Jinju-si Gyeongsangnam-do, 52727 Republic of Korea |
| Yong Sok Ji | 1725 | Chonnam National University Hospital 42, Jebong-ro, Donggu Gwangju, 61469 Republic of Korea |
| Kang Yeun Pak | 1726 | lnje University Haeundae Paik Hospital 875 Heaundae-ro Heaundae-gu Busan, 48108 Republic of Korea |
| Young Hoon Lee | 1727 | Konyang University Hospital 158, Gwanjeodong-ro, Seo-gu Daejeon, 35365 Republic of Korea |
| **Bulgaria** |  |  |
| Christina Grupcheva | 2001 | Specialized ophthalmology hospital for active treatment - Varna EOOD 15 Doyran Street Varna, 9002 Bulgaria |
| Iva Petkova | 2002 | Specialized hospital for active treatment of eye diseases Zora OOD Mladost district, 4 Petar Protich Street Sofia, 1784 Bulgaria |
| Alexander Oscar | 2003 | Diagnostic-consultative center Aleksandrovska EOOD, 1 Sveti Georgi Sofiyski Street Sofia, 1431 Bulgaria |
| Botio Anguelov | 2004 | Multiprofile Hospital for Active TreatmentSveta Sofia EOOD Department of ophthalmology diseases 104 Bulgaria Boulevard Sofia, 1404 Bulgaria |
| **Czech Republic** |  |  |
| Vladimir Korda | 3001 | OFTEX s.r.o. Rokycanova 2798 Pardubice, 53002 Czech Republic |
| Bohdan Kousal | 3002 | Všeobecná fakultní nemocnice v Praze, Oční klinika U Nemocnice 2 Praha 2, 128 00 Czech Republic |
| **Hungary** |  |  |
| Andras Papp | 4001 | Semmelweis Egyetem, Szemészeti Klinika Mária utca 41 Budapest, 1085 Hungary |
| Balázs Varsanyi | 4002 | Ganglion Medical Center Varadi Antal utca 10/A Pécs, 7621 Hungary |
| Norbert Czumbel | 4003 | Jahn Ferenc Dél-pesti Kórház es Rendelóintézet, Szemészeti Osztály Köves utca 1 Budapest, 1204 Hungary |
| Attila Vajas | 4005 | Debreceni Egyetem Klinikai Központ, Szemészeti Klinika Nagyerdei krt 98 Debrecen, 4032 Hungary |
| Alexis Tsorbatzoglou | 4006 | Szabolcs-Szatmár-Bereg Megyei Kórházak és Egyetemi Oktatókórház Jósa András Oktatókórház Szemszéti Osztály Szent István u. 68 Nyiregyhaza, 4400 Hungary |
| András Seres | 4007 | Budapest Retina Associates Kft. Váci út 76. 2. torony Ill.em Budapest, 1133 Hungary |
| Gábor Vogt | 4008 | Magyar Honvédség Egészségugyi Központ, Szemeszéti Osztály Dózsa Gyorgy út 112. D. ép. 7.em Budapest, 1062 Hungary |
| **India** |  |  |
| Anup Kelgaonkar | 5001 | L V Prasad Eye Institute MTC Campus, Patia Bhubaneswar 751024, Odisha India |
| Sachin Vishwanath Daigavane | 5002 | Acharya Vinoba Bhave Rural Hospital Jawaharlal Nehru Medical College Datta Meghe Institute of Medical Sciences Sawangi (M) Wardha 442004, Maharashtra India |
| Asim Kumar Ghosh | 5003 | Regional Institute of Ophthalmology 88, College Street Kolkata 700073, West Bengal India |
| Arti Elhence | 5004 | Sam Eye Hospital 21/31 Tilak Marg (Off-Rana Pratap Marg) Hazratganj Lucknow 226001, Uttar Pradesh India |
| Shahana Mazumdar  Anubhav Goyal | 5006 | ICare Eye Hospital & Post Graduate Institute E-3A, Sector - 26 Noida 201301, Uttar Pradesh India |
| Smiti Rani Srivastava | 5010 | Institute of Post Graduate Medical Education and Research and SSKM Hospital 244, AJC Bose Road Kolkata 700020, West Bengal India |
| Virendra Agrawal | 5011 | Dr. Virendra Laser Phaco Surgery Centre Tonk Road, TonkPhatak Gandhi Nagar Jaipur 302015, Rajasthan India |
| **Israel** |  |  |
| Yoreh Barak | 6001 | Rambam Medical Center Ophthalmology Department, 8 Haaliya Hashniya Street Haifa 31096 Israel |
| Eva Eting | 6002 | Institutional Helsinki Committee Shamir Medical Center Assaf Harofeh Beer Yaakov Tzrifin 70300 Israel |
| Tareq Jaouni | 6003 | Hadassah Medical Center Opthalmology Department PO Box 12-221 Jerusalem 91120 Israel |
| Iris Moroz | 6004 | The Chaim Sheba Medical Center Ophthalmology Department Tel-Hashomer Ramat Gan 5262100 Israel |
| Nurit Mathalone | 6006 | Carmel Medical Center Ophthalmology Department Horev 2 Haifa 34362 Israel |
| Haia Morori-Katz | 6007 | Kaplan Medical Center Ophthalmology Department Pasternak Street Post Office Box 1 Rehovot 76100 Israel |
| Nakhoul Nakhoul | 6008 | Baruch Padeh Poriya Medical Center Ophthalmology Department M.p. Lower Galilee Tiberias 15208 Israel |
| Irit Rosenblatt | 6009 | Rabin Medical Center Ophthalmology Department 39 Jabotinsky Street Petach Tikva 49100 Israel |
| Alexander Rubowitz | 6010 | Meir Medical Center Ophthalmology Department 59 Tchernichovsky Street Kfar Saba 44281 Israel |
| Oksana Gagarin | 6011 | Bnai Zion Medical Center Ophthalmology Department 47 Golomb Street Haifa 31048 Israel |
| Dinah Zur | 6012 | Tel Aviv Sourasky Medical Center Ophthalmology Department 6 Weitzman Street Tel Aviv 64239 Israel |
| Asaf Bar | 6013 | Edith Wolfson Medical Center Ophthalmology Department 62 Halohamim Street Holon 58100 Israel |
| Avraham Cohen | 6014 | Galilee Medical Center POB 21 Nahariya 22100 Israel |
| **Japan** |  |  |
| Hiroshi Enaida | 8001 | Saga University Hospital 1-1, 5-chome, Nabeshima Saga-shi Saga 849-8501 Japan |
| Takatoshi Maeno | 8002 | Toho University Sakura Medical Center 564-1 Shimoshizu Sakura Chiba 285 8741 Japan |
| Miki Sawa | 8003 | Sakai City Medical Center 1-1-1 Ebaraji-cho, Nishi-ku Sakai-Shi Osaka 593 8304 Japan |
| Tetsuju Sekiryu | 8004 | Fukushima Medical University Hospital 1 Hikarigaoka Fukushima City Fukushima 960-1295 Japan |
| Toru Noda | 8005 | National Hospital Organization Tokyo Medical Center 2-5-1, Higashigaoka Meguro-ku 152-8902 Japan |
| Kazuhiro Kimura | 8006 | Yamaguchi University Hospital 1-1-1, MinamiKogushi, Ube City Yamaguchi Prefecture 755-8505 Japan |
| Fumi Gomi | 8007 | Hyogo College of Medicine College Hospital 1-1, Mukogawacho Nishinomiya Hyogo 663-8501 Japan |
| Taiji Sakamoto | 8008 | Kagoshima University Hospital 8-35-1 Sakuragaoka Kagoshima 890-8520 Japan |
| Ken Hayashi | 8009 | Hayashi Eye Hospital 4-23-35 Hakataekimae Hakata-ku Fukuoka 812-0011 Japan |
| Tatsushi Kaga | 8010 | Japan Community Health care Organization (JCHO) Chukyo Hospital 1-1-10, Sanjo, Minami-ku Nagoya Aichi 457-8510 Japan |
| Kanako Yasuda | 8011 | Tokyo Medical University Hachioji Medical Center 1163, Tate-machi Hachioji-shi Tokyo 193-0998 Japan |
| Hideyasu Oh | 8013 | Hyogo Prefectural Amagasaki General Medical Center 2-17-77 Higashinaniwacho Amagasaki Hyogo 660-8550 Japan |
| Masatoshi Haruta | 8015 | Kurume University Hospital 67 Asahi-machi Kurume Fukuoka 830-0011 Japan |
| Hideki Koizumi | 8016 | University of the Ryukyus Hospital 207 Uehara Nakagamigun Nishiharacho Okinawa 903-0215 Japan |
| Yoko Ozawa | 8017 | St. Luke's International Hospital 9-1 Akashi-cho Chuo-ku Tokyo 104 8560 Japan |
| Kiyoshi Suzuma | 8018 | Kagawa University Hospital 1750-1 Ikenobe, Miki-cho Kita-gun Kagawa Prefecture 761-0793 Japan |
| Kyoko Fujita | 8019 | Aichi Medical University Hospital 1-1 Yazakokarimata Nagakute Aichi 480-1195 Japan |
| Noriko Miyamoto | 8020 | Rakuwakai Otowa Hospital 2 Otowachinji-cho Yamashina-ku Kyoto 607-8062 Japan |
| Hiroko Terashima  Hiruma Hasebe | 8021 | Niigata University Medical and Dental Hospital 754, Ichibancho Asahimachidori, Chuo-ku Niigata-shi Niigata 951-8520 Japan |
| Yasuhiko Hirami | 8022 | Kobe City Eye Hospital 2-1-8 Minatojima Minamimachi Chuo-ku, Kobe-city Hyogo 650-0047 Japan |
| Toshiyuki Yokoyama | 8023 | Juntendo University Nerima Hospital 3-1-10, Takanodai Nerima-ku Tokyo 177-8521 Japan |
| Atushi Hayashi | 8024 | Toyama University Hospital 2630, Sugitani Toyama 930-0194 Japan |
| **Latvia** |  |  |
| Indars Lacis | 9001 | Latvian American Eye Center Augusta Deglava Street 12a Riga LV-1009 Latvia |
| Guna Laganovska | 9002 | Pauls Stradins Clinical University Hospital Pilsonu Street 13 Riga LV-1002 Latvia |
| Kristine Baumane | 9003 | Riga Eastern Clinical University Hospital, Clinic Bikernieki Lielvardes 68 Riga LV-1006 Latvia |
| Signe Ozolina | 9004 | Signes Ozolinas Doctor Praxis In Ophthalmology Ikveza O. Kalpaka Street 16 Jelgava LV-3001 Latvia |

**e-Appendix 2.** List of independent ethics committees and institutional review boards

Australia

Bellberry Human Research Ethics Committee

St. Vincent's Hospital Melbourne Human Research Ethics Committee

Poland

Komisja Bioetyczna przy Okregowej Izbie Lekarskiej w Gdańsku

Russia

The Russian Federation Ministry of Healthcare Department of State Regulation of Circulation of Medicines Ethics Council

Ethics Committee at Federal State Autonomous Institution “National Medical Research Centre” “Interdisciplinary Scientific and Technical Complex” “Microsurgery of the Eye" n. a. “S.N. Fedorov” of the Ministry of Health of the Russian Federation

The Russian Federation Ministry of Healthcare Department of State Regulation of Circulation of Medicines Ethics Council

“Moscow State Medical Stomatology University n. a. A.I. Evdokimov” of the Ministry of Health of the Russian Federation

Ethics Committee at Federal State Budgetary Institution “Helmholtz National Medical Research Centre of Eye Diseases” of the Ministry of Health of the Russian Federation

Ethics Committee at State Budgetary Healthcare Institution of Novosibirsk Region “State Novosibirsk Regional Clinical Hospital”, Ophthalmology Department

Slovakia

Etická komisia Univerzitnej nemocnice Bratislava Nemocnica Ružinov

Eticka komisia Nemocnica Poprad, a.s.

Spain

CEIm Hospital Puerta de Hierro

USA

Advarra IRB

South Korea

Asan Medical Center Institutional Review Board

Ajou University Hospital Institutional Review Board

Pusan National University Hospital Institutional Review Board

SMG - SNU Boramae Medical Center Institutional Review Board

Keimyung University Dongsan Hospital Institutional Review Board

Yeungnam University Hospital Institutional Review Board

Kyungpook National University Hospital Institutional Review Board

Inha University Hospital Institutional Review Board

Samsung Medical Center IRB

Kyung Hee University Hospital Institutional Review Board

Gyeongsang National University Changwon Hospital IRB

Hanyang University Guri Hospital Institutional Review Board

Seoul National University Hospital Institutional Review Board

Nune Eye Hospital

Hallym University Kangdong Sacred Heart Hospital Institutional Review Board

Dong-A University Hospital Institutional Review Board

Kosin University Gospel Hospital Institutional Review Board

Chungnam National University Hospital Institutional Review Board

Korea University Anam Hospital Institutional Review Board

Chungbuk National University Hospital Institutional Review Board

Gachon University Gil Medical Center Institutional Review Board

Gyeongsang National University Hospital Institutional Review Board

Chonnam National University Hospital, Institutional Review Board

Inje University Haeundae Paik Hospital Institutional Review Board

Konyang University Hospital

Bulgaria

Ethics Committee for Clinical Trials

Czech Republic

Etická komise Fakultní nemocnice Hradec Králové

Eticka komise VFN v Praze, Na Bojisti

Hungary

Egészségügyi Tudományos Tanács Klinikai Farmakológiai Etikai Bizottság

Országos Gyógyszerészeties Élelmezés-egészségügyi Intézet

India

Institutional Ethics Committee L V Prasad Eye Institute

Institutional Ethics Committee Datta Meghe Institute of Medical Sciences (DU)

Institutional Ethics Committee, Regional Institute of Ophthalmology

Institutional Ethics Committee For Sam Eye Hospital

ICare Eye Hospital & Post Graduate Institute Ethics Committee

IPGMER Research Oversight Committee

Institutional Ethics Committee Dr. Virendra Laser Phaco Surgery Centre

Israel

Institutional Helsinki Committee Rambam Medical Center

Institutional Helsinki Committee Shamir Medical Center Assaf Harofeh

Institutional Helsinki Committee Hadassah Medical Center

Institutional Helsinki Committee The Chaim Sheba Medical Center

Institutional Helsinki Committee Carmel Medical Center

Institutional Helsinki Committee Kaplan Medical Center

Institutional Helsinki Committee Baruch Padeh Poriya Medical Center

Institutional Helsinki Committee Rabin Medical Center

Institutional Helsinki Committee Meir Medical Center

Institutional Helsinki Committee Bnai Zion Medical Center

Institutional Helsinki Committee Tel Aviv Sourasky Medical Center

Institutional Helsinki Committee Edith Wolfson Medical Center

Institutional Helsinki Committee Galilee Medical Center

Japan

Saga University Hospital IRB

Toho University Sakura Medical Center IRB

Sakai City Medical Center Institutional Review Board

Haradoi Hospital Institutional Review Board

National Hospital Organization Tokyo Medical Center IRB

Yamaguchi University Hospital IRB

Hyogo College of Medicine College Hospital Institutional Review Board

Kagoshima University Hospital IRB

Hayashi Eye Hospital Institutional Review Board

Japan Community Health Care Organization (JCHO) Chukyo Hospital IRB

Tokyo Medical University Hachioji Medical Center Institutional Review Board

Hyogo Prefectural Amagasaki General Medical Center IRB

Yokohama Minoru Clinic Institutional Review Board

University of the Ryukyus Hospital IRB

St. Luke's International Hospital IRB

Kagawa University Hospital IRB

Aichi Medical University Hospital IRB

Rakuwakai Otowa Hospital Institutional Review Board

Niigata University Medical and Dental Hospital IRB

Kobe City Medical Center General Hospital IRB

Juntendo University Nerima Hospital IRB

Toyama University Hospital Drug Acceptance Research Review Board

Latvia

The Ethics Committee for Clinical Trials of Medicinal Products

**e-Appendix 3.** Full List of Inclusion and Exclusion Criteria

**Inclusion Criteria**

Subjects were eligible for inclusion if they met all of the following main criteria:

1. Subject was ≥50 years of age.

2. Subject had active choroidal subfoveal, juxtafoveal, or extrafoveal neovascularization lesions secondary to age-related macular degeneration (AMD) evidenced by fluorescein angiography (FA) in the study eye at screening and confirmed by the central reading center.

3. Subject had the best corrected visual acuity (BCVA) letter score of 73 to 35 using original series Early Treatment Diabetic Retinopathy Study (ETDRS) charts or 2702 series number charts in the study eye at screening and Week 0 (Day 1) prior to randomization. In addition, the fellow eye was not to have less than 35 letter score using the ETDRS chart or 2702 series number chart.

**Exclusion Criteria**

Subjects were excluded from participation in this study if they met any of the following main criteria:

1. Subject had any prior ocular (in the study eye and fellow eye) or systemic treatment or surgery for neovascular AMD except dietary supplements or vitamins.

2. Subject had any prior or concomitant therapy with another investigational agent to treat neovascular AMD in the study eye, except dietary supplements or vitamins.

3. Subject’s fellow eye showed signs of AMD that, in investigator’s medical opinion, needed any treatment during the study period.

4. Subject had received any prior treatment with anti-VEGF agents in both eyes (ie, completely treatment naïve subjects only were to be included).

5. The total lesion size was >30.5 mm2, including blood, scars, atrophy, fibrosis, and neovascularization as assessed by FA in the study eye and confirmed by the central reading center.

6. Subject had central retinal thickness (CRT) of <300 μm in the study eye and was confirmed by the central reading center.

7. Subject had a subretinal hemorrhage that was either 50% or more of the total lesion area or if the blood was under the fovea and was 1 or more disc areas in size in the study eye and was confirmed by the central reading center (if the blood was under the fovea, then the fovea had to be surrounded 270 degrees by visible CNV).

8. Subject had scar or fibrosis, making up >50% of the total lesion in the study eye and confirmed by the central reading center.

9. Subject had scar, fibrosis, or atrophy involving the center of the fovea in the study eye and confirmed by the central reading center.

10. Subject had retinal pigment epithelial tears or rips involving the macula in the study eye and confirmed by the central reading center.

11. Subject had Lens Opacity Classification System II Grade IV cataract in the study eye, or other significant cataract in the study eye that in the investigator’s opinion interfered with visualization of the retina or interfered with retinal imaging.

12. Subject had active intraocular/periocular infection and inflammation in either eye.

13. Subject had a history of any vitreous hemorrhage in the study eye within 4 weeks prior to the Screening Visit.

14. Subject had other causes of CNV in the study eye as confirmed by the central reading center.

15. Subject had a history or clinical evidence of diabetic retinopathy, diabetic macular edema, or any other vascular disease affecting the retina, other than AMD, in either eye.

16. Subject had prior vitrectomy in the study eye.

17. Subject had a history of retinal detachment, treatment, or surgery for retinal detachment in the study eye.

18. Subject had a history of macular hole of Stage 2 and above in the study eye as confirmed by the central reading center.

19. Subject had a history of uncomplicated intraocular or periocular surgery within 3 months of Day 1 on the study eye, except lid surgery, which may not have taken place within 1 month of Day 1. Note: A subject with uncomplicated neodymium yttrium aluminum garnet laser capsulotomy performed for secondary opacification of

the posterior capsule in intraocular lens implanted eye within 3 months prior to Day 1 in the study eye was to be considered as eligible.

20. Subject had aphakia in the study eye.

21. Subject had a history of glaucoma-filtering surgery within 3 months of Day 1 in the study eye. Anti-glaucoma laser surgeries were not to be considered exclusionary.

22. Subject had a history of corneal transplant in the study eye.

23. Subject had a history or evidence of any other clinically significant disorder, condition, or disease (eg, co-existence of retinal vein occlusion, radiation retinopathy, diabetic retinopathy, glaucoma under treatment) in the study eye that, in the opinion of the investigator, would pose a risk to subject safety or interfere with the study evaluation, procedure or complication.

24. Subject had a history of laser therapy in the macular region in the study eye.

25. Subject had any prior or concomitant treatment with intravitreal corticosteroids injection, intravitreal corticosteroid implant, subtenon corticosteroids, or peribulbar corticosteroids in the study eye 6 months before the Screening Visit. Note: For intravitreal corticosteroid implant, the exclusion period was to be 36 months from the date of the procedure to the date of screening.

26. Subject had any prior or concomitant treatment involving the macula with photodynamic therapy with verteporfin, transpupillary thermotherapy, radiation therapy, or retinal laser treatment (eg, focal laser photocoagulation) in the study eye.

27. Subject had any prior or concomitant treatment with pan-retinal photocoagulation in the study eye 90 days before the Screening Visit.

28. Subject had any concomitant or prior treatment with ethambutol (2 weeks prior to randomization); deferoxamine and topiramate (4 weeks prior to randomization); tamoxifen, hydroxychloroquine, chloroquine, or vigabatrin (8 weeks prior to randomization), and amiodarone (12 weeks prior to randomization).

29. Subject had any investigational product for the treatment of ocular conditions (in either eye) and systemic conditions 30 days or 5 half-lives (whichever is longer) prior to randomization and throughout the study, except dietary supplements or vitamins.

30. Subject had intraocular pressure (IOP) ≥25 mmHg in spite of anti-glaucoma treatment.

31. Subject had any prior or ongoing systemic medical condition (including but not limited to infectious, inflammatory, psychiatric, neurological, renal, hepatic, respiratory conditions, or malignancies) or clinically significant screening laboratory value that in the opinion of the investigator presented a safety risk, interfered with study compliance and follow-up, or confounded data interpretation throughout the study period.

**eTable 1.** Incidence of Antidrug Antibody (ADA) and Neutralizing Antibody (Nab) Positivity at Each Visit

| **Visit** | **Parameter** | **SCD411 (N = 287) n/N (%)** | **Aflibercept (N = 286) n/N (%)** | **Total (N = 573) n/N (%)** |
| --- | --- | --- | --- | --- |
| **Baseline** | ADA | 20/281 (7.1) | 19/277 (6.9) | 39/558 (7.0) |
|  | NAb | 3/281 (1.1) | 6/277 (2.2) | 9/558 (1.6) |
| **Week 4** | ADA | 80/272 (29.4) | 100/272 (36.8) | 180/544 (33.1) |
|  | NAb | 6/272 (2.2) | 10/272 (3.7) | 16/544 (2.9) |
| **Week 8** | ADA | 108/270 (40.0) | 147/281 (52.3) | 255/551 (46.3) |
|  | NAb | 5/271 (1.8) | 9/281 (3.2) | 14/552 (2.5) |
| **Week 20** | ADA | 103/260 (39.6) | 106/266 (39.8) | 209/526 (39.7) |
|  | NAb | 3/260 (1.2) | 3/266 (1.1) | 6/526 (1.1) |
| **Week 36** | ADA | 58/253 (22.9) | 60/257 (23.3) | 118/510 (23.1) |
|  | NAb | 2/253 (0.8) | 5/254 (2.0) | 7/507 (1.4) |
| **Week 52** | ADA | 51/252 (20.2) | 48/255 (18.8) | 99/507 (19.5) |
|  | NAb | 2/244 (0.8) | 7/250 (2.8) | 9/494 (1.8) |

ADA, antidrug antibody; Nab, neutralizing antibody; n, number of subjects with positive result; N, number of subjects available for assessing result at each visit

* ADA concentration with signal above cut point of 10.0 ng/mL is defined as ADA positive

* If ADA result is negative, then the Nab result is considered as negative

**eFigure 1.** Change from Baseline in BCVA score for Study Eye by Visit

BCVA, best-corrected visual acuity; n, number

**eFigure2.** Change form Baseline in CRT for Study Eye by Visit

CRT, central retinal thickness; n, number

**eFigure 3.** The Mean Free Plasma Concentration versus Time Profiles for SCD411 and Aflibercept


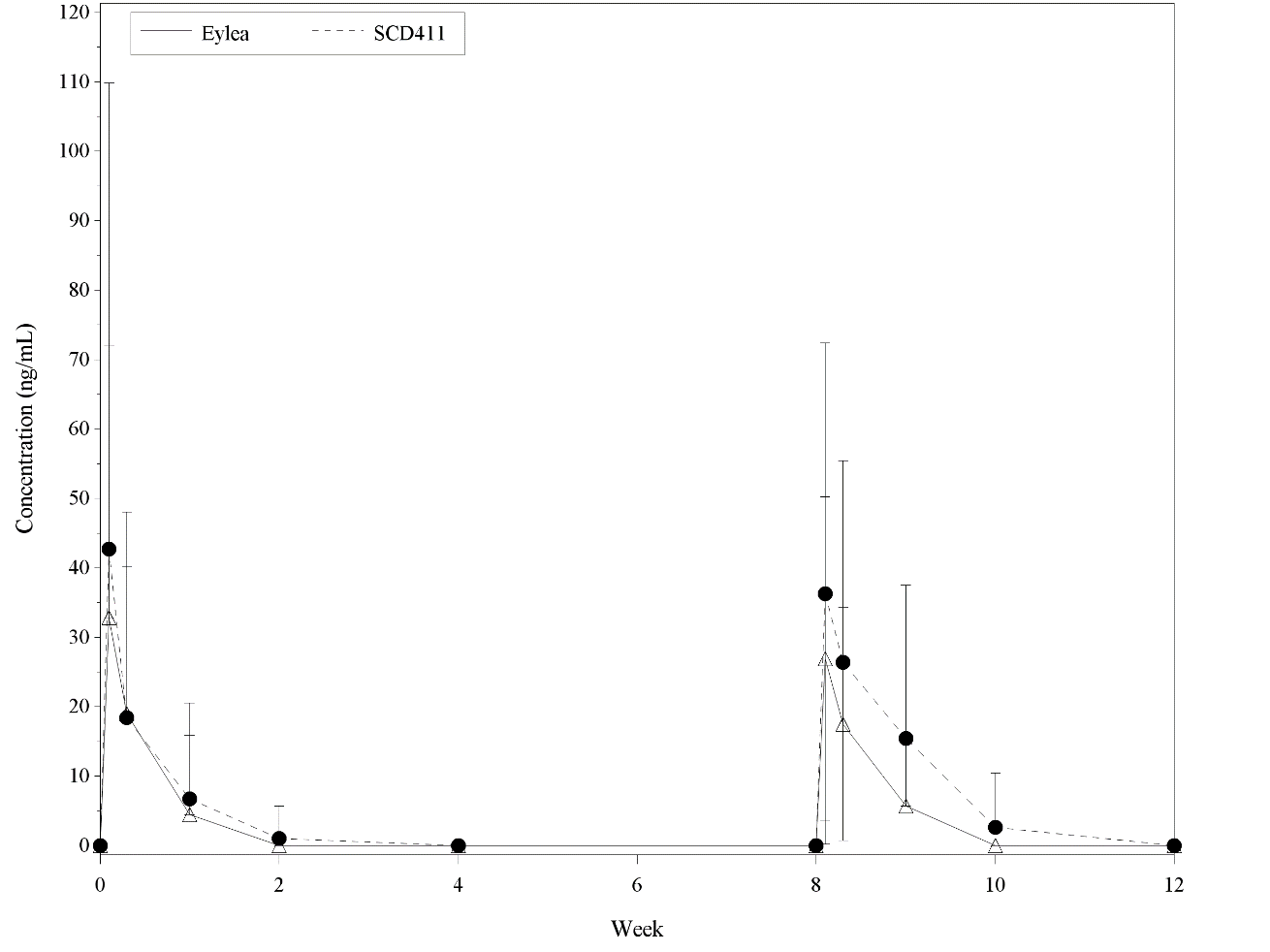

Supplement: Supplementary file 2 — Supplementary Information 2. [file 41598_2024_65815_MOESM2_ESM.docx]
