# Supplementary material for: Comparison of the efficacy and safety of SCD411 and reference aflibercept in patients with neovascular age-related macular degeneration
Source: Sci Rep. 2024 Jun 26;14:14752. doi: 10.1038/s41598-024-65815-6 (PMC11208512; doi:10.1038/s41598-024-65815-6)
Supplement: Supplementary file 3 — Supplementary Information 3. [file 41598_2024_65815_MOESM3_ESM.pdf]

Protocol Synopsis

**Protocol Number:**	SCD411-CP101
**Title:**	A Phase III Randomized, Double-Masked, Parallel-Group, Multicenter Study to Compare the Efficacy, Safety, Tolerability, Pharmacokinetics, and Immunogenicity between SCD411 and Eylea® in Subjects with Neovascular Age-related Macular Degeneration
**Sponsor:**	SamChunDang Pharm. Co. Ltd.<br>351, Hyoryeong-ro, Seocho-gu, Seoul 06643, Republic of Korea
**Study Phase:**	Phase III
**Study Sites:**	Approximately 155 study sites and 14 countries
**Indication:**	Neovascular Age-related Macular Degeneration
**Rationale:**	SCD411 is being developed as a biosimilar to the reference product Eylea® (aflibercept). Based on the proposed extensive analytical comparability testing, comparative nonclinical testing, and comparative clinical studies using the same licensed reference product, the current study is designed to evaluate comparability between SCD411 and aflibercept. Based on the data from aflibercept clinical studies VIEW 1, VIEW 2, COPERNICUS, GALILEO, VIBRANT, VIVID, VISTA, and MYRROR, wet age-related macular degeneration (AMD) is considered as the most suitable condition to prove similarity between SCD411 and aflibercept as the reference product.<br>The available data regarding the pharmacokinetics (PK) of the reference product suggests that a proper PK characterization will not be possible after single intravitreal (IVT) administration. In a PK substudy of Eylea in 6 neovascular wet AMD patients with frequent sampling, maximum plasma concentrations (C_{max}) of free aflibercept (systemic) were low, with a mean of approximately 0.02 µg/mL (range 0 to 0.054) within 1 to 3 days after a 2 mg IVT injection and were undetectable 2 weeks following dosage in almost all patients. It is evident that in some cases no measurable systemic levels of free aflibercept were registered after IVT administration. Hence, a comparative PK evaluation in patients with AMD will likely not provide any information of relevance to support biosimilarity. Although the systemic exposure of SCD411 is expected to also be very low at steady state, there is no previous human data on SCD411, and thus this assumption requires experimental support. Hence, PK assessment will be performed in the current study following the first and the third doses of SCD411.

As aflibercept is a therapeutic protein, there is a potential for immunogenicity. No cases of active neutralizing antibodies (NAb) were observed after administration of Eylea. It is evident that immunogenicity is not presenting a substantial safety concern in patients treated with aflibercept injections. There was no substantial difference in the proportion of patients testing positive for anti-drug antibodies (ADA) in different indications, including wet AMD. Based on this observation, the indication chosen for this study is expected to provide sound data for comparing the immunogenicity of the test product (SCD411) with that of Eylea.

The clinical part of the comparison will be based on clinical efficacy and safety data obtained after IVT administration to subjects with wet AMD in the current pivotal study.

Objectives:

The primary objective of this study is:

- To prove the equivalence of SCD411 as compared to Eylea (aflibercept) in best corrected visual acuity (BCVA) after 8 weeks of treatment among subjects with wet AMD.

The secondary objectives of this study are:

- To compare the safety and tolerability of SCD411 and aflibercept
- To compare the efficacy of SCD411 and aflibercept after 8 weeks and 52 weeks of treatment demonstrated by BCVA, central retinal thickness (CRT), and choroidal neovascularization (CNV)
- To compare the immunogenicity of SCD411 and aflibercept by presenting information of the development of anti-SCD411 antibodies.

The exploratory objectives of this study are:

- To compare PK parameters of SCD411 and aflibercept
- To quantify free and bound aflibercept and SCD411.

Estimands:

The primary estimand for the primary objective is the mean treatment difference in BCVA change from baseline at Week 8. At Week 52, the primary estimand of interest is the mean treatment difference in BCVA change from baseline at Week 52. For the primary estimand, any observations post receipt of rescue therapy in the study eye will be set to missing, thereby estimating the treatment effect as though subjects do not receive rescue therapy. For each of the Week 8 and Week 52 analyses, the secondary estimand will be presented where post rescue will not be set to missing but will be included in the mixed-effects model for repeated measures

	(MMRM) analysis. The secondary estimands are constructed to estimate the treatment effect while including the impact of rescue therapy, which may better reflect actual real-world outcomes, where rescue therapy would likely be given when lack of efficacy is seen.
**Study Population:**	Each subject must meet all of the following inclusion criteria to be enrolled in this study:<br>1. Capable of understanding the written informed consent, provides signed and witnessed written informed consent, and agrees to comply with protocol requirements<br>2. Age ≥50 years<br>3. Active choroidal subfoveal, juxtafoveal, or extrafoveal neovascularization lesions secondary to AMD evidenced by fluorescein angiography (FA) in the study eye at screening and confirmed by the central reading center<br>4. The BCVA letter score of 73 to 35 using original series Early Treatment Diabetic Retinopathy Study (ETDRS) charts or 2702 series number charts in the study eye at screening and at Week 0 (Day 1) prior to randomization. In addition, fellow eye should not be less than 35 letter score using the ETDRS chart or 2702 series number chart<br>5. Women of child-bearing potential with a negative serum pregnancy test at screening must agree to use protocol-defined methods of contraception throughout the study until 3 months after the last injection of aflibercept/SCD411<br>6. Males with female partners of child-bearing potential must agree to use protocol-defined methods of contraception and agree to refrain from donating sperm throughout the study until 3 months after the last injection of aflibercept/SCD411.<br>7. The area of CNV making up either 50% or more of the total lesion area and confirmed by the central reading center<br><br>Subjects meeting any of the following criteria at the Screening Visit will be excluded from the study:<br>1. Any prior ocular (in the study eye and fellow eye) or systemic treatment or surgery for neovascular AMD except dietary supplements or vitamins<br>2. Any prior or concomitant therapy with another investigational agent to treat neovascular AMD in the study eye, except dietary supplements or vitamins

3. Fellow eye shows signs of AMD that, in investigator's medical opinion, may need any treatment during study period
4. Any prior treatment with anti-vascular endothelial growth factor (VEGF) agents in the both eyes (ie, completely treatment naïve subjects only to be included)
5. Total lesion size >30.5 mm^2, including blood, scars, atrophy, fibrosis, and neovascularization as assessed by FA in the study eye and confirmed by the central reading center
6. Central retina thickness of <300 μm in the study eye and confirmed by the central reading center
7. Subretinal hemorrhage that is either 50% or more of the total lesion area, or if the blood is under the fovea and is 1 or more disc areas in size in the study eye and confirmed by the central reading center. (If the blood is under the fovea, then the fovea must be surrounded 270 degrees by visible CNV.)
8. Scar or fibrosis, making up >50% of the total lesion in the study eye and confirmed by the central reading center
9. Scar, fibrosis, or atrophy involving the center of the fovea in the study eye and confirmed by the central reading center
10. Presence of retinal pigment epithelial tears or rips involving the macula in the study eye and confirmed by the central reading center
11. Lens Opacity Classification System II (LOCS II) grade IV cataract in the study eye, or other significant cataract in the study eye that in the Investigator's opinion interferes with visualization of retina or interferes with retinal imaging
12. Active intraocular/periocular infection and inflammation in either eye
13. History of any vitreous hemorrhage in the study eye within 4 weeks prior to the Screening Visit
14. Presence of other causes of CNV in the study eye as confirmed by central reading center
15. History or clinical evidence of diabetic retinopathy, diabetic macular edema, or any other vascular disease affecting the retina, other than AMD, in either eye
16. Prior vitrectomy in the study eye
17. History of retinal detachment, treatment, or surgery for retinal detachment in the study eye
18. History of macular hole of Stage 2 and above in the study eye as confirmed by central reading center

19. History of uncomplicated intraocular or periocular surgery within 3 months of Day 1 on the study eye, except lid surgery, which may not have taken place within 1 month of Day 1

Note: A subject with uncomplicated neodymium yttrium aluminum garnet (Nd:YAG) laser capsulotomy performed for secondary opacification of the posterior capsule in intraocular lens implanted eye within 3 months prior to Day 1 in the study eye will be considered as eligible.

20. Presence of aphakia in the study eye.

21. History of glaucoma-filtering surgery within 3 months of Day 1 in the study eye. Anti-glaucoma laser surgeries will not be considered exclusionary.

22. History of corneal transplant in the study eye.

23. History or evidence of any other clinically significant disorder, condition or disease (eg, co-existence of retinal vein occlusion, radiation retinopathy, diabetic retinopathy, glaucoma under treatment) in the study eye that, in the opinion of the investigator, would pose a risk to subject safety or interfere with the study evaluation, procedure or complication

24. Uncontrolled hypertension defined as systolic blood pressure (BP) >160 mmHg or diastolic BP >100 mmHg under appropriate antihypertensive treatment

25. Hypersensitivity to aflibercept or medications used in this study (fluorescein, mydriatic eye drops, etc.)

26. Pregnancy or lactation at the Screening Visit and/or at baseline for women of child-bearing potential

27. Any contraindication to IVT injection according to the investigator’s clinical judgment

28. History of thrombotic events (eg, stroke, transient ischemic attacks, pulmonary embolism, deep vein thrombosis, or myocardial infarction)

29. History or evidence of cardiac conditions with congestive cardiac failure resulting in marked limitation on physical activity or inability to perform any physical activity without discomfort; subjects with ventricular arrhythmia requiring ongoing treatment; or subjects with atrial fibrillation

30. History of laser therapy in the macular region in the study eye

31. Any prior or concomitant treatment with IVT corticosteroids injection, IVT corticosteroid implant, subtenon corticosteroids, or

	peribulbar corticosteroids in the study eye 6 months before the Screening Visit<br>Note: For IVT corticosteroid implant, the exclusion period would be 36 months from the date of the procedure to the date of screening<br>32. Any prior or concomitant treatment involving the macula with photodynamic therapy with verteporfin, transpupillary thermotherapy, radiation therapy, or retinal laser treatment (eg, focal laser photocoagulation) in the study eye<br>33. Any prior or concomitant treatment with pan-retinal photocoagulation 90 days in the study eye before the Screening Visit<br>34. Any concomitant or prior treatment with ethambutol (2 weeks prior to randomization); deferoxamine and topiramate (4 weeks prior to randomization); tamoxifen, hydroxychloroquine, chloroquine, or vigabatrin (8 weeks prior to randomization), and amiodarone (12 weeks prior to randomization)<br>35. Any investigational product for the treatment of ocular conditions (in either eye) and systemic conditions 30 days or 5 half-lives (whichever is longer), prior to randomization, and throughout the study, except dietary supplements or vitamins<br>36. Intraocular pressure ≥25 mmHg in spite of anti-glaucoma treatment<br>37. Any prior or ongoing systemic medical condition (including but not limited to infectious, inflammatory, psychiatric, neurological, renal, hepatic, respiratory conditions or malignancies) or clinically significant screening laboratory value that in the opinion of the investigator may present a safety risk, interfere with study compliance and follow-up, or confound data interpretation throughout the study period.
**Study Design:**	This is a Phase III, randomized, double-masked, parallel-group, multicenter study to demonstrate biosimilarity of SCD411 compared to Eylea (aflibercept) among adult subjects with neovascular (wet) AMD. Subjects will be randomly assigned in 1:1 ratio using Interactive Response Technology to receive either SCD411 or aflibercept injections. Subjects will receive their randomized first IVT injection on Day 1 and subsequent IVT injection on Weeks 4, 8, 16, 24, 32, 40, and 48. The End-of-Treatment (EOT) Visit will be scheduled for Week 48. Subjects who discontinue the study treatment early should have the reasons for treatment discontinuation documented on the Treatment Discontinuation page of the electronic case report form (eCRF). Treatment discontinuation in the study is defined as subjects discontinuing the

	study treatment due to adverse event/lack of efficacy/rescue treatment but not limited to these conditions. Every effort should be made to have such subjects remain in the study and continue with their regularly scheduled visits and assessments until Week 52 except for study treatment administration. If a subject chooses to withdraw from the study or if, in the opinion of the investigator, the subject should be prematurely withdrawn from the study, then the Early Termination (ET) Visit should be completed as soon as possible after discontinuing the study treatment but no later than 28 days after discontinuation. The End-of-Study (EOS) Visit will occur 28 days after the EOT at Week 52. Subjects and study site staff involved in subject management and study assessments will be masked to study treatment assignment. The investigator involved in performing the IVT injections will be unmasked to study treatment.
**Estimated Study Duration:**	The total study duration is approximately 55 weeks, including a screening duration up to 3 weeks, treatment duration up to 48 weeks, and a follow-up duration up to 4 weeks.
**Efficacy Assessments:**	Efficacy will be summarized for the study eye only.<br>Primary endpoint:<br>• Change from baseline in BCVA as measured by ETDRS letters score or 2702 charts at Week 8.<br>Secondary endpoints:<br>• Change from baseline in BCVA as measured by ETDRS letter score or 2702 charts at Week 52<br>• Change from baseline in CRT at Week 8 as assessed by optical coherence tomography (OCT)<br>• Change from baseline in CRT at Week 52 as assessed by OCT<br>• Change from baseline in CNV area at Week 8<br>• Change from baseline in CNV area at Week 52<br>• Percentage of subjects who gain at least 15 letters in BCVA as measured by ETDRS letter score or 2702 charts compared with baseline at Week 8<br>• Percentage of subjects who gain at least 15 letters in BCVA as measured by ETDRS letter score or 2702 charts compared with baseline at Week 52.
**Pharmacokinetic Assessments:**	Pharmacokinetic parameters include area under the concentration-time curve from time zero to the last quantifiable time point (AUC_{0-t}), AUC from zero to the end of the dosing period (AUC_{0-tau}), AUC from zero to infinite time (AUC_{0-inf}), C_{max}, time to reach maximum

	plasma concentration (t_{max}), and elimination half-life ($t_{1/2}$), of SCD411 and aflibercept. Quantification of free and bound aflibercept and SCD411 in plasma will be performed using blood samples at predose and at +1 day, +3 days, +7 days, +14 days, and +28 days after the first (Day 1) and third (Week 8) doses.
**Immunogenicity Assessments:**	Immunogenicity assessments and endpoints include the evaluation of development of anti-SCD411 or anti-aflibercept antibodies using blood samples taken at Baseline, at Weeks 4, 8, 20, 36, and 52. The samples will be evaluated for titer of anti-SCD411 or anti-aflibercept antibodies.<br>Neutralizing antibodies will be tested when ADA results are confirmed to be positive.
**Safety Assessments:**	Safety endpoints include adverse events (AEs), vital signs, and laboratory assessments up to Week 52. Safety is also evaluated during follow-up (4 weeks after EOT). If a subject is discontinued from the study, all the ET Visit procedures should be performed even if they are outside the allowed study window.
**Study Drug, Dosage, and Route of Administration:**	One IVT injection containing 2 mg dose of either SCD411 or aflibercept (as per randomization) every 4 weeks for 3 consecutive doses, followed by 1 injection every 8 weeks.
**Rescue Treatment**	In the study eye, rescue treatment for neovascular AMD is not permitted at any point in time. If it is observed that efficacy is not achieved by the study treatment, as determined by the investigator, subjects can be considered for rescue treatment after Week 4 if any 1 of the following conditions are met as assessed by the masked investigators:<br>• Decrease of visual acuity letter score of 15 letters or more from the last assessment and/or<br>• An increase in intraretinal fluid, subretinal fluid, or subretinal hyper-reflective material, that in the investigator's opinion is related to progression of the subject's neovascular AMD.<br>Subjects who will receive rescue treatment need to discontinue the study treatment and should have the reason for treatment discontinuation documented on the Treatment Discontinuation page of the eCRF and continue with their regularly scheduled visits and assessments until Week 52 except for study treatment administration. If a subject chooses to withdraw from the study or if, in the opinion of the investigator, the subject should be prematurely withdrawn from the study, then the ET Visit should be completed as

	soon as possible after discontinuing the study treatment but no later than 28 days after discontinuation.
**Sample Size:**	The equivalence margin agreed upon with European Medicines Agency (EMA) and Pharmaceuticals and Medical Devices Agency (PMDA) was determined using the data presented from MARINA (and ANCHOR) and VIEW studies. The data were used to estimate the efficacy of aflibercept compared to the placebo treatment in subjects with wet AMD. It was shown that in MARINA, subjects on average lost 2.5 BCVA letters over 2 months in the sham-treated control arm and gained 5 letters in the treatment arm of 2 mg dose, and that in the VIEW studies 6.8 to 7.5 letters were gained among subjects with wet AMD at Week 8 into treatment on a 2 mg dose. Combining both study data, the treatment effect compared to the placebo would be estimated as approximately 9.3 to 10 letters. A margin of 3.8 letters would be translated to a preservation of 59% to 62% of the difference between Eylea and sham and is less than a 1 line change in visual acuity. Equivalence discussions for EMA and PMDA were based upon a 95% confidence interval (CI) approach or 2 one-sided tests (TOST) at the $\alpha=0.025$ level.<br><br>The US Food and Drug Administration (FDA) requested a tighter equivalence margin of 3 letters but agreed equivalence can be determined from a 90% CI approach (equivalent to TOST at the $\alpha=0.05$ level).<br><br>Standard deviation (SD) for the mean change from baseline at early visits (Weeks 4 to 12) based on observed data for the full analysis set (FAS) ranged from and 8.73 to 11.99 considering all treatment groups from VIEW1 and from 7.4 to 11.0 letters for the VIEW2 study. For power calculations, a range of SD between 10.4 and 11.8 was assumed to be conservative and cover the majority of larger SD values seen in the observed data.<br><br>A sample size of 266 subjects per treatment arm was selected as it provides at least 80% power for the FDA, EMA, and PMDA analyses for the range of SD considered when using TOST on data from a parallel-group design. For the FDA analysis based on equivalence limits of -3.0 and 3.0 letters, $\alpha=0.05$ significance level (90% CI), assuming the true difference between the means is 0.0, power of 91% is achieved for SD of 10.4 letters and power of 80% is achieved for SD of 11.8 letters. For the EMA and PMDA analyses based on equivalence limits of -3.8 and 3.8 letters, $\alpha=0.025$ significance level (95% CI), assuming the true difference between the means is 0.0, power of 98% is achieved for SD of 10.4 letters and power of 92% is achieved for SD of 11.8 letters when sample

	size is 266 per treatment arm. Considering approximately 5% loss from randomization through Week 8, the total sample size required is 560.<br>A subset of 40 subjects (20 per group) will be selected for collection of PK samples. The sample size is not test-driven since no equivalence test will be performed for PK parameters.
**Analysis Sets**	The following analysis sets will be used in the statistical analyses:<br>**Full Analysis Set (FAS):** All randomized subjects who received at least 1 injection of the study drug.<br>**Modified FAS (mFAS)**: All randomized subjects who received at least 1 injection of the study drug and had at least 1 postbaseline BCVA assessment in the study eye.<br>**Per-protocol Set (PPS):** All subjects in the FAS, excluding those with significant protocol violations.<br>**Safety Set (SAF):** All subjects receiving at least 1 injection of the study drug.<br>**Pharmacokinetic Analysis Set (PK Set):** The subset of subjects in FAS who have sufficient evaluable blood samples to be included in the PK Set.<br>The primary set for efficacy analysis will be the FAS; however, for the EMA submission, the primary efficacy endpoint must meet equivalence for both the FAS and PPS. PMDA also requires an efficacy analysis to be conducted based on the mFAS without multiple imputation (MI) as a supportive analysis. The SAF will be the primary analysis set for safety, tolerability, and immunogenicity analyses.<br>Efficacy will be assessed for the study eye only.
**Statistical Methods:**	**Primary Analysis:**<br>The primary analysis of the change from baseline in BCVA as measured by ETDRS letter score or 2702 charts through 8 weeks of treatment will be performed via MMRM including data for the Week 4 and Week 8 visits. The model will include the change from baseline as the dependent variable; treatment, visit, and visit-by-treatment as fixed effects, and baseline BCVA as a covariate. Equivalence will be determined if the upper and lower CI limits for the difference between treatments are within the equivalence margins.<br>The primary null hypothesis (H0) and alternative hypothesis (H1) are:<br>H0: $\Delta \geq M$ or $\Delta \leq -M$

H1: -M< Δ <M

where Δ indicates the mean difference between the 2 treatment groups in the change from baseline in ETDRS letter score or 2702 charts at Week 8 and M represents the equivalence margin.

Based on discussions with regulatory agencies, separate equivalence CIs and equivalence margins will be used for FDA submissions and other regulatory agencies versus submissions to EMA and PMDA.

For the FDA submission and other regulatory agencies, a 90% CI (equivalent to TOST evaluated at an α of 0.05) will be assessed using an equivalence margin of 3 letters. For the EMA and PMDA, a 95% CI (equivalent to TOST evaluated at an α of 0.025) will be assessed using an equivalence margin of 3.8 letters. Other regulatory submissions are assumed to follow the FDA guidance.

Estimates of the treatment difference and corresponding 90% (FDA and other countries) and 95% (EMA/PMDA) CIs will be provided. The primary analysis set for the FDA, PMDA, and any other submissions will be the FAS. For the EMA submission, both the FAS and PPS are considered primary analysis and equivalence will need to be shown for both analysis sets. The analysis will be performed using multiple imputed data under the missing at random (MAR) assumption. The primary analysis will set post rescue assessments to missing prior to the MI procedure.

Secondary Analyses:

The estimated mean treatment difference in the change from baseline in BCVA as measured by ETDRS letter score or 2702 charts through 52 weeks along with the corresponding 90% and 95% CIs from the same MMRM model as used in the primary analysis but including data from all weeks through Week 52 will also be presented for the FAS and PPS. As done for Week 8, analysis will be performed using multiple imputed data, where post rescue assessments have been set to missing while including the post rescue assessments in the imputation model. Raw values and changes from baseline will be summarized by treatment group using descriptive statistics for each visit.

The analysis of change from baseline in CRT as assessed by OCT or change from baseline in CNV will be based on the MMRM model similar to the one used for the analysis of change from baseline in BCVA, with the corresponding 95% CI for the estimates of treatment differences at Week 8 and Week 52 presented. Raw values and changes from baseline will be summarized by treatment group

using descriptive statistics. Data after the receipt of rescue therapy will be set to missing prior to the MMRM analysis. Multiple imputation will not be performed, and no sensitivity analyses will be performed.

The percentage of subjects who gain ≥15 letters in BCVA as measured by ETDRS letter score or 2702 charts at postbaseline visits (Week 8 and Week 52) compared with baseline will be summarized and 95% CI for the proportions in each treatment arm and difference in proportions between treatment arms will be presented. For subjects who have missing assessments at corresponding postbaseline visits or who received rescue therapy prior to a visit will be assumed to have a gain of <15 letters in BCVA (eg, nonresponder imputation). No sensitivity analyses will be performed.

Safety Analyses

An overall summary of AEs will be presented, and treatment-emergent AEs will be summarized by system organ class and preferred term. Results of the following safety evaluations will be summarized with observed values and change from baseline: vital signs, ECGs, laboratory examinations including chemistry, hematology, and urinalysis. The results of slit lamp examination, dilated fundoscopy, intraocular pressure, and vision check will be summarized using descriptive statistics at each visit and applicable time point, as applicable.

Pharmacokinetic Analysis

The PK parameters ($AUC_{0\text{-}t}$, $AUC_{0\text{-}tau}$, $AUC_{0\text{-}inf}$, C_{max}, t_{max}, $t_{1/2}$) from the 2 treatment groups will be summarized using n, arithmetic mean, percent coefficient of variation (% CV), geometric mean, geometric %CV, SD, median, minimum and maximum values in the PK analysis set.

Immunogenicity Analysis

The analysis on immunogenicity will be descriptive. Results will be summarized with observed values and change from baseline for ADA titers (in log scale) by treatment group in the SAF. The number and proportion of ADA-positive subjects and ADA-negative subjects will be summarized by treatment group for each assessment time point. NAb will be tested when ADA results are confirmed to

	be positive. The number and proportion of NAb subjects will be summarized by treatment group for each assessment time point.<br><br>**Interim Analyses**<br>An interim analysis of safety, secondary efficacy endpoints, and PK parameters will be performed once approximately 200 subjects complete the Week 52 Visit and all subjects complete the Week 24 Visit or discontinue early from the study.<br>The Week 8 analysis and interim analysis will be performed by an independent biostatistics group and results will be distributed to a limited group of recipients, including unmasked medical writing staff and limited sponsor representatives. The timing of the Week 8 database lock and the interim analysis database lock may coincide depending on subject enrollment and data cleaning/programming activities.
**Version and Date of Protocol:**	Version 3.0<br>24 January 2022

3.2 Protocol Amendments and Other Changes in the Conduct of the Study

3.2.1 Changes in the Conduct of the Study

Two major amendments and 2 country-specific (Slovakia- and Korea-specific) amendments were made to the original protocol, dated 27 Mar 2020 and implemented. A country-specific amendment for Italy was released on 24 Jul 2020 but no subjects were recruited under this amendment.

The following is a summary of the major changes implemented with Protocol Amendment 1, Version 2.0, dated 24 Nov 2020:

- Section 2.5 Estimands: This section was revised as per the United States Food and Drug Administration (US FDA) requirement of changing the Full Analysis Set (FAS) and the recommendation of not discontinuing subjects from the study when they discontinue study treatment.
- Section 3.1 Study Design:
    - As per the request from the Israel regulatory agency, it was clarified that subjects in Israel will not participate in the PK substudy.
    - Stratification by subjects in Japan was added to the protocol as per the sponsor's decision.
    - The paragraph on study treatment discontinuation was updated as per the US FDA recommendation to minimize missing data by keeping subjects who discontinue study treatment in the study to continue with regularly scheduled visits, so that their efficacy and safety data after treatment discontinuation could be collected to support sensitivity analyses.
    - Figure 3-1 Study Schema was updated to remove the laboratory assessments at the Week 48 EOT Visit.
- Section 4.1.1 Inclusion Criteria:
    - Inclusion criterion 3 was updated by adding the range of CNV lesions as per the request from the Korean Ministry of Food and Drug Safety

(MFDS), which had been incorporated into the Korea-specific protocol amendment.

- Inclusion criterion 7 was added as per the request from the Korean MFDS to clarify that CNV should be either 50% or more of the total lesion area in the inclusion criterion. This change had been applied to the Korea-specific protocol amendment.

- Section 4.1.2 Exclusion Criteria:
    - Exclusion criterion 12 was updated to clarify that subjects with the conditions of intraocular/periocular infection and inflammation in either eye were to be excluded at Screening. This change had been applied to the Korea-specific protocol amendment.
    - The limit of systolic blood pressure was decreased from 180 to 160 mmHg in exclusion criterion 24, as per the request from the Slovakia regulatory agency. This change had been applied to the Slovakia-specific protocol amendment.
    - As per the requests from the Slovakia regulatory agency and the Korean MFDS, exclusion criterion 36 regarding the intraocular pressure (IOP) condition in spite of anti-glaucoma treatment was added according to the Summary of Product Characteristics. This change had been applied to both Slovakia- and Korea-specific protocol amendments.
    - As per the request from the Slovakia regulatory agency, subjects with a systemic medical condition were to be excluded from the study; therefore, exclusion criterion 37 was added.
- A new Section 4.2 Selection of Study Eye was added as per the US FDA requirement to clearly define the study eye in the protocol.
- Section 4.3.1 Discontinuation From Study Treatment and Section 4.3.2 Withdrawal From the Study:
    - As per the US FDA recommendation to keep subjects who discontinued study treatment in the study to continue with their regularly scheduled visits, the reasons for withdrawal/discontinuation were listed separately.

  - For clarity, certain specific criteria for treatment discontinuation were moved to the section of discontinuation of study treatment.

- Section 4.3.3 Handling of Withdrawals: The section was updated as per the US FDA recommendation to keep subjects who discontinue study treatment in the study and continue with their regularly scheduled visits.
- Section 4.3.4 Screen Failures: Text was revised to clarify that if an image retransmission was requested from the central image center vendor due to any possible technical issues, this was not to be considered rescreening.
- Section 5.2 Treatments Administered: To further ensure the safety of subjects, the condition of pre-injection IOP was added and clarified in the protocol as per the request from the Russian regulatory agency. To avoid duplicated post-injection assessments of IOP and dilated fundoscopy by the masked and unmasked staff, both assessments were removed for the unmasked staff and were only to be conducted by the masked staff. The sponsor confirmed that the hand movement/finger counting checks by the unmasked team were sufficient for safety assessment.
- Section 5.2.1 Treatment of Fellow Eye: This section was revised as per the sponsor's decision to allow treatment of fellow eye only with Eylea for the whole duration of the study.
- Table 5-1: The VEGF treatment was updated as a prohibited medication only applicable to the study eye. Additionally, the note on treatment of fellow eye was updated to reflect that it should be treated only with Eylea.
- Section 5.3 Identity of IP: As per the US FDA request, the source of Eylea was updated.
- Section 5.8.1 Rescue Treatment:
  - This section was updated as per the US FDA recommendation to let subjects who discontinued study treatment due to receipt of rescue treatment in the study to continue with their regularly scheduled visits.
  - Due to the sponsor's concern on the potential error caused by the IOP device, the condition for rescue treatment of an 'increase in the central subfield thickness of 100 μm compared with the latest assessment (optical coherence tomography [OCT]) by the investigator' was removed.

- Section 5.8.2 Prohibited Medications: Prohibited medications applied to the fellow eye were removed as per the sponsor's decision to allow fellow eye treatment only with Eylea for the whole duration of the study.
- Section 6.1.3 Early Termination/End-of-Study: This section was updated as per the US FDA request to keep subjects who discontinued study treatment in the study to continue with their regularly scheduled visits.
- Section 6.3.3.6 Suspected Unexpected Serious Adverse Reactions and Nonserious Adverse Events of Special Interest (AESIs): The title of the section and the body text on nonserious AESIs were removed because there is no definition of AESI in the protocol.
- Section 6.4 Pharmacokinetic Assessments: A sentence was added to clarify that additional visits were to be arranged for collecting PK blood samples.
- Section 6.7 Pregnancy: This section was updated to clarify that the serum pregnancy test was to be administered only to women of childbearing potential and to implement the change to keep subjects in the study to continue regularly scheduled visits and assessments after study treatment discontinuation. Additionally, follow-up for safety until the outcome of the pregnancy was known was emphasized.
- Table 6-1 Schedule of Events: The footnotes to the Schedule of Events were updated to reflect changes in the text of the protocol.
- Table 6-2 Clinical Laboratory Evaluations: The table of clinical laboratory evaluations was updated to include all the laboratory tests provided by the central laboratory.
- Section 7.1 Estimands and Intercurrent Events: The section on estimands and intercurrent events was updated as per the US FDA request to change the FAS population and to not discontinue subjects from the study when they discontinued study treatment due to AE/lack of efficacy/rescue treatment.
- Section 7.2 Sample Size Determination: Justification for the 3.8 letter equivalence margin was mentioned.
- Section 7.3 Analysis Sets: The definition of the FAS was updated as per the request from US FDA. The definitions of the Safety Set and the PK Set were updated for clarity.

- Section 7.5.1.1 Primary Efficacy Outcome Measures: The section of primary efficacy outcome measures was updated as per the US FDA request to change the FAS population and to not discontinue subjects from the study when they discontinued study treatment due to AE/lack of efficacy/rescue treatment. The covariance structure was specified as per the request from US FDA.
- Section 7.5.1.2 Secondary Efficacy Outcome Measures: The section on secondary efficacy outcome measures was updated as per the US FDA request to change the FAS population and to not discontinue subjects from the study when they discontinued study treatment due to AE/lack of efficacy/rescue treatment.
- Section 11.2.2 Protocol Deviations: This section was updated as per the request from the Korean MFDS to list serious protocol violations in the protocol. The update had been applied to the Korea-specific protocol amendments.
- Section 12 Reference List: The reference list was updated with the European Medicines Agency's (EMA) overview of Eylea.

The following is a summary of the major changes implemented with Protocol Amendment 2, Version 3.0, dated 24 Jan 2022:

- Exclusion criterion 29 (Synopsis; Section 4.1.2) for subjects with a history or evidence of cardiac conditions was reworded for clarity.
- Statistical methods (Synopsis; Section 3.1 Study Design; Section 7 Statistical Analytical Plan; Section 7.5.6 Interim Analyses): Interim analysis was added to support regulatory filing to the Pharmaceutical and Medical Devices Agency (PMDA).
- Section 6.3.3.1 Definitions of Adverse Events; Section 6.3.3.4 Reporting Adverse Events; Section 6.3.3.8 Assessment of Causality: An assessment of AEs was included for the IVT injection procedure.
- Section 6.6 Independent Data Monitoring Committee; Section 11.1.1 External Data Monitoring Committee; Section 11.4 Final Report: Text was modified to reflect the addition of an interim analysis.
- Throughout the protocol: Changes were made to achieve consistency between different sections of the protocol and statistical analysis plan (SAP) and to improve the readability and overall quality of the document.

Schedule of Events

Assessment	Screening	Treatment													ET[a]/EOS[b]
**Study Day**	**–21 to 0**	**Baseline/1**	**29**	**57**	**85**	**113**	**141**	**169**	**197**	**225**	**253**	**281**	**309**	**EOT/337**	**365**
**Study Week**	**-3**	**1**	**4**	**8**	**12**	**16**	**20**	**24**	**28**	**32**	**36**	**40**	**44**	**48**	**52**
**Visit Time Window (days)**			**±2**	**±2**	**±7**	**±7**	**±7**	**±7**	**±7**	**±7**	**±7**	**±7**	**±7**	**±7**	**±7**
**Study Visit**	**1**	**2**	**3**	**4**	**5**	**6**	**7**	**8**	**9**	**10**	**11**	**12**	**13**	**14**	**15**
Informed consent	X														
Demographic information (incl. height and weight)	X														
Inclusion/exclusion criteria	X	X													
Ocular and systemic medical history	X														
Slit lamp examination[c]	X	X	X	X	X	X	X	X	X	X	X	X	X	X	X
Dilated fundoscopy[d]	X	X	X	X	X	X	X	X	X	X	X	X	X	X	X
Pregnancy test[e]	X	X													X
Prior and concomitant ocular and systemic medications[f]	X	X	X	X	X	X	X	X	X	X	X	X	X	X	X
Randomization[g]		X													
SCD411 or aflibercept injection [h]		X	X	X		X		X		X		X		X	
Pre-injection IOP[i]	X	X	X	X	X	X	X	X	X	X	X	X	X	X	X
Post-injection IOP and vision check[i]		X	X	X		X		X		X		X		X	
BCVA[j]	X	X	X	X	X	X	X	X	X	X	X	X	X	X	X
OCT[k]	X	X	X	X	X	X	X	X	X	X	X	X	X	X	X
FA and FP[l]	X			X											X
Laboratory assessments[m]	X	X	X	X			X								X
Vital signs[n]	X	X	X	X	X	X	X	X	X	X	X	X	X	X	X
ECG	X			X											X

Assessment	Screening	Treatment													ET[a]/EOS[b]
**Study Day**	**–21 to 0**	**Baseline/1**	**29**	**57**	**85**	**113**	**141**	**169**	**197**	**225**	**253**	**281**	**309**	**EOT/337**	**365**
**Study Week**	**-3**	**1**	**4**	**8**	**12**	**16**	**20**	**24**	**28**	**32**	**36**	**40**	**44**	**48**	**52**
**Visit Time Window (days)**			**±2**	**±2**	**±7**	**±7**	**±7**	**±7**	**±7**	**±7**	**±7**	**±7**	**±7**	**±7**	**±7**
**Study Visit**	**1**	**2**	**3**	**4**	**5**	**6**	**7**	**8**	**9**	**10**	**11**	**12**	**13**	**14**	**15**
AEs	X	X	X	X	X	X	X	X	X	X	X	X	X	X	X
Immunogenicity samples[o]		X	X	X			X				X				X
PK samples[p]		X		X											

Abbreviations: AEs, adverse events; BCVA, best corrected visual acuity; ECG, electrocardiogram; EOS, end-of-study; EOT, end-of-treatment; ET, early termination; FA, fluorescein angiography; FP, fundus photography; FSH, follicle-stimulating hormone; IOP, intraocular pressure; IVT, intravitreal; LH, luteinizing hormone; OCT, optical coherence tomography; PDT, photodynamic therapy; PK, pharmacokinetics; SAE, serious adverse event; VA, visual acuity; VEGF, vascular endothelial growth factor.

[a] For subjects who discontinue study treatment and choose to withdraw from the study or if, in the opinion of the investigator, the subject should be prematurely withdrawn from the study, ET Visit assessments are to be performed as soon as possible after discontinuing the study treatment but no later than 28 days after discontinuation.

[b] End-of-study assessments are to be performed 28 days after the EOT.

[c] Slit lamp examination will be performed on both eyes at all the planned scheduled visits during the study.

[d] Dilated fundoscopy will be performed on the study eye at all the planned scheduled visits during the study, including the time prior to IVT injection of the study drug, and within 60±30 minutes after IVT injection of the study drug, and at any time during the visit at ET/EOS Visit.

[e] Serum pregnancy test will be done at screening, and urine pregnancy test at baseline, ET, and EOS Visits for female subjects of child-bearing potential. If urine pregnancy test is positive, serum pregnancy test should be performed; if found positive, study treatment will be discontinued, and the subject should continue with their regularly scheduled visits and assessments until Week 52 except for study treatment administration. Pregnancy cases should be followed until resolution of the pregnancy.

[f] Concurrent use of systemic or intravitreal anti-VEGF agents, IVT, subtenon, or peribulbar corticosteroids in study eye, except as required to treat AEs, and use of photocoagulation or PDT with verteporfin are prohibited during the study. Antimicrobial drops can be used at the discretion of the investigator. For IVT corticosteroid implant, the exclusion period would be 36 months from the date of the procedure to the date of screening.

[g] The investigator must confirm that the subject meets all inclusion and none of the exclusion criteria at both screening and Day 1. This is inclusive of BCVA scoring.

[h] Rescue treatment for the study eye is not permitted at any point in time. Subjects can be considered for rescue treatment after Week 4 if the following conditions are met as assessed by the masked investigator: VA letter score decrease of 15 letters or more from the last assessment, an increase in intraretinal fluid, subretinal fluid, or subretinal hyper-reflective material, that in the investigator’s opinion is related to progression of the subject’s neovascular AMD. Subjects who will receive rescue treatment need to discontinue the study treatment and should have the reason for treatment discontinuation documented on the Treatment Discontinuation page of the eCRF and continue with all their regularly scheduled visits and assessments until Week 52 except for study treatment administration. If a subject chooses to withdraw from the study or if, in the opinion of the investigator, the subject should be prematurely withdrawn from the study, then the ET Visit should be completed as soon as possible after discontinuing the study treatment but no later than 28 days after discontinuation.
